# Co‐Delivery of Sustained Release Chondroitinase ABC‐37 With Human iPSC‐Derived Neural Progenitors Promotes Transplant Survival and Functional Recovery in a Rodent Model of Stroke

**DOI:** 10.1002/advs.202522499

**Published:** 2026-09-09

**Authors:** Nitzan Letko Khait, David Xinzheyang Li, Keyi Guo, Hong Cui, Quinton Sirianni, Dhana Abdo, Cindi M. Morshead, Molly S. Shoichet

**Affiliations:** ^1^ Department of Chemical Engineering and Applied Chemistry University of Toronto Toronto Ontario Canada; ^2^ Donnelly Centre University of Toronto Toronto Ontario Canada; ^3^ Institute of Biomedical Engineering University of Toronto Toronto Ontario Canada; ^4^ Department of Surgery University of Toronto Toronto Ontario Canada; ^5^ Department of Chemistry University of Toronto Toronto Ontario Canada

**Keywords:** chondroitinase ABC, hyaluronic acid, hydrogel, neural progenitor cells, stroke

## Abstract

Stroke is a leading cause of disability worldwide, yet effective treatments to regenerate damaged tissue remain elusive. We investigated tissue regeneration and functional recovery post‐stroke in an animal model with a combinatorial approach leveraging human induced pluripotent stem cell‐derived neural progenitor cells (NPCs) and a sustained‐release formulation of a re‐designed, thermostable chondroitinase ABC (ChASE37). We optimized an in situ gelling, injectable hyaluronic acid (HA)‐based hydrogel with the incorporation of laminin to deliver NPCs to the cavity in a rat model of sub‐acute ischemic stroke. We delivered ChASE37 by affinity release from a methylcellulose hydrogel (ChASE37‐AR) to the stroke‐injured brain by epicortical injection. ChASE37‐AR retained long‐term enzymatic activity, as demonstrated by degradation of inhibitory chondroitin sulfate proteoglycans (CSPGs) in the injured tissue, with no evidence of immunogenicity. Each treatment — ChASE37‐AR, NPCs, and their co‐delivery — significantly improved motor function as early as 3 weeks after a single administration. Critically, only the combined therapy supported long‐term survival and neuronal differentiation of transplanted cells, suggesting that ChASE37‐AR has a role beyond CSPG degradation. This strategy presents a modular, clinically relevant platform for tissue regeneration post‐stroke. Together, these findings suggest that the co‐delivery of NPCs and sustained‐release ChASE37 may be promising for stroke treatment.

## Graphical abstract

A regenerative strategy for stroke combines human stem cell–derived neural progenitor cells with sustained release of a matrix‐modifying, thermostable chondroitinase ABC‐37 enzyme. In a rat model of stroke, co‐delivery enhanced transplanted cell survival and neuronal differentiation, degraded inhibitory extracellular matrix components, and improved motor recovery. This modular hydrogel‐based platform offers a clinically relevant approach to post‐stroke tissue repair.

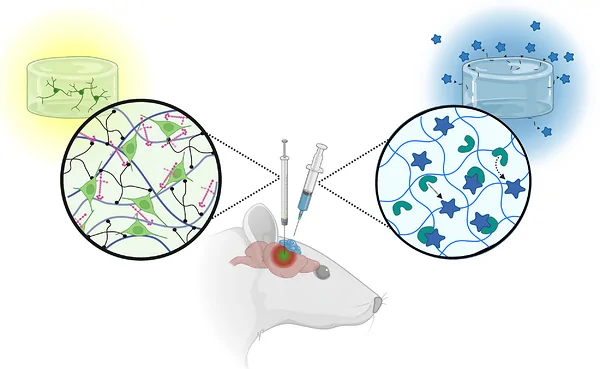

## 1 Introduction

Stroke remains the second leading cause of mortality and the third leading cause of long‐term disability globally, with approximately half of all stroke survivors experiencing chronic disabilities [1]. Ischemic stroke, the most common subtype, results from a severe reduction in oxygen and nutrient supply to brain tissue. This deprivation triggers neuronal cell death, disrupts the blood‐brain barrier (BBB), and initiates a cascade of inflammatory responses. Consequently, a cavity forms at the injury site, surrounded by a reactive astrocytic border, the glial scar, that further hinders neural repair [2, 3, 4]. Currently, the only FDA‐approved treatments for acute ischemic stroke aim to restore blood flow to the affected brain region. These include intravenous tissue plasminogen activator (tPA) and mechanical thrombectomy, both of which focus on removing the occlusion. However, these interventions are limited by a narrow therapeutic time window, leaving over 85% of stroke patients reliant on rehabilitation therapies alone [5, 6, 7]. No treatments replace lost neuronal cells or reestablish structural and functional integrity in the damaged tissue.

Cell‐based therapies promise to regenerate lost tissue and enhance functional outcomes after stroke, as has been thoroughly described in multiple review articles [8, 9, 10]. The application of neural progenitor cells (NPCs) derived from human induced pluripotent stem cells (iPSC) holds significant therapeutic potential because the NPCs can differentiate into mature neurons, astrocytes, and oligodendrocytes, potentially replacing lost neural populations, and/or secrete a broad spectrum of trophic factors that enhance endogenous repair processes within the host tissue [11, 12, 13]. Notwithstanding significant advancements in recent years, ensuring graft survival and integration post‐transplantation remain major challenges [12, 14].

Biomaterial scaffolds have been shown to enhance cell viability during injection and provide a supportive framework within the tissue [15, 16]. Injectable, soft materials, such as hydrogels, offer several advantages, including the ability to mimic the native microenvironment of the brain [4]. Hydrogels can stimulate key processes such as proliferation and neuronal differentiation by fine‐tuning material stiffness, degradation rate, and hydrogel remodeling, as well as incorporating biologically relevant cues, like brain‐abundant glycosaminoglycans and proteins [17].

Hyaluronic acid (HA), a naturally occurring non‐sulfated glycosaminoglycan, is a major component of the brain's extracellular matrix (ECM). HA plays a critical role in various biological processes including cell adhesion, migration, proliferation, differentiation, inflammation, angiogenesis, and tissue regeneration [18, 19]. These functions are mediated primarily through interactions with CD44, a transmembrane receptor expressed on the surface of most cell types. HA is versatile: it is biodegradable, chemically modifiable, and possesses tunable mechanical properties to support various cell types. Consequently, HA‐based hydrogels have been extensively studied for their potential to facilitate cell and drug delivery to the brain [20, 21, 22, 23].

Perineuronal nets (PNNs) surround neurons and are mainly comprised of chondroitin sulfate proteoglycans (CSPGs) and an HA backbone. CSPGs are critical for PNN structure and function, and act through their core protein and covalently attached sulfated glycosaminoglycan (GAG) chains. These sulfated disaccharide units confer a negative charge, enabling the binding of various proteins that interact with neuronal surface receptors [24]. CSPGs can also directly engage cell surface receptors. Most CSPGs in the CNS are found in the diffuse extracellular matrix and are less densely organized than those in the PNNs [25].

Recent studies have underscored the importance of PNNs in regulating neural plasticity. Following CNS injury, such as stroke or spinal cord injury (SCI), a CSPG‐rich glial scar forms at the lesion site in both animal models and humans [26]. This scar inhibits axonal regeneration and presents a key hurdle to both transplanted cell survival and endogenous repair [27]; yet, astrocytes within the scar retain phenotypic plasticity and respond to local signaling cues that can alter their functional properties [28, 29]. The scar thickens and matures over time, starting to form hours after the injury, for at least 14 d [30]. During the acute phase post‐injury, CSPGs limit the extent of tissue degeneration to adjacent healthy tissue; however, in the chronic phase of recovery, CSPGs form a significant barrier to axonal sprouting and tissue regeneration, thereby impeding long‐term repair and functional recovery [31, 32]. To address this challenge, scar prevention and/or CSPG degradation have been pursued to enhance repair [27, 33, 34].

Chondroitinase ABC (ChASE) and its re‐engineered analog, ChASE37, are bacterial enzymes that degrade CSPGs and have demonstrated efficacy in preclinical models of stroke and SCI [35, 36, 37, 38, 39, 40]. ChASE37 (i.e., with 37 point mutations) can be delivered locally from an affinity‐controlled release (AR) system: reversible interactions between a fusion protein of Src homology‐3 (SH3)‐ChASE37 and SH3‐binding peptides covalently bound to a crosslinked methylcellulose hydrogel enable sustained release [41].

**FIGURE 1:**
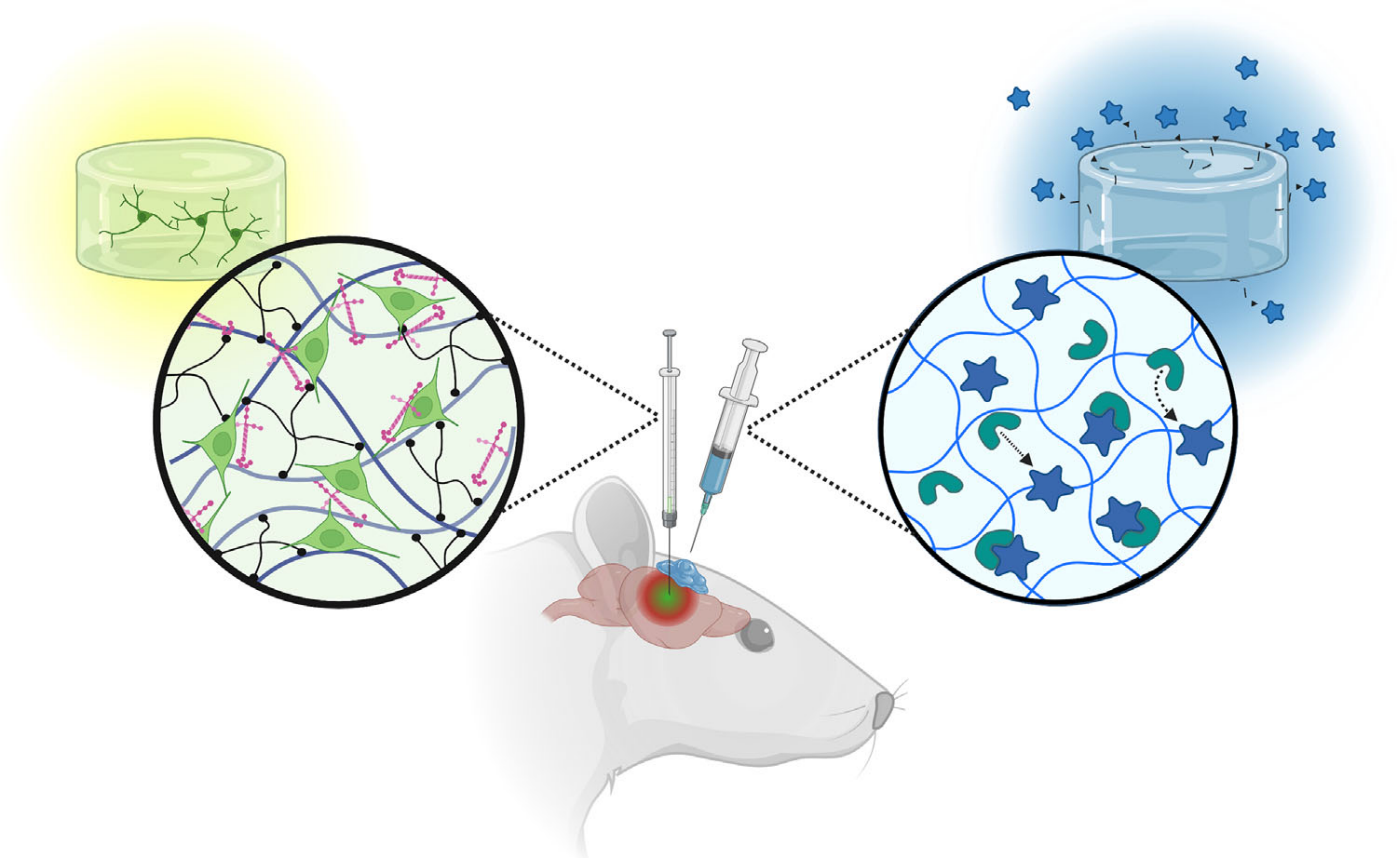
Combined delivery of neural progenitor cells (NPCs) and chondroitinase ABC 37 (ChASE37) to overcome stroke. NPCs were implanted into the lesion cavity using a hyaluronan‐based hydrogel to support survival and integration, while thermostabilized ChASE37 was applied epicortically using a methylcellulose hydrogel from which it diffused into the brain to modulate the inhibitory extracellular matrix.

We wondered if the combination of NPC cell therapy and ChASE37‐AR (as illustrated in Figure 1) would promote tissue and functional repair after stroke. We first investigated NPC survival and differentiation in HA‐based hydrogels and found that encapsulated NPCs differentiated to neurons in vitro. To evaluate the efficacy of our combinatory therapy, we used the endothelin‐1 (ET‐1) model of stroke, which closely mimics moderate human ischemic stroke, characterized by a rapid reduction in blood flow followed by slow reperfusion [42]. Notably, ET‐1‐injured rodents exhibited cavity formation surrounded by astrogliosis and CSPG deposition in the peri‐infarct zone, resembling human stroke pathology [26, 43]. Using the ET‐1 model, we tested each treatment alone and in combination and found that all treated animals had significantly enhanced functional recovery relative to vehicle controls. ChASE37‐AR remained bioactive, as shown by degradation of CSPGs in and around the lesion site 4 weeks following treatment. Interestingly, surviving transplanted cells were only observed in animals treated with both NPCs and ChASE37‐AR, suggesting an indirect effect of ChASE37‐specific CSPG degradation on NPC survival. The surviving cells displayed early neuronal phenotypes, demonstrating the potential of the co‐treatment to repopulate stroke‐damaged tissue and enhance recovery.

## 2 Results

### 2.1 Neural Progenitor Cell Differentiation and Characterization

**FIGURE 2:**
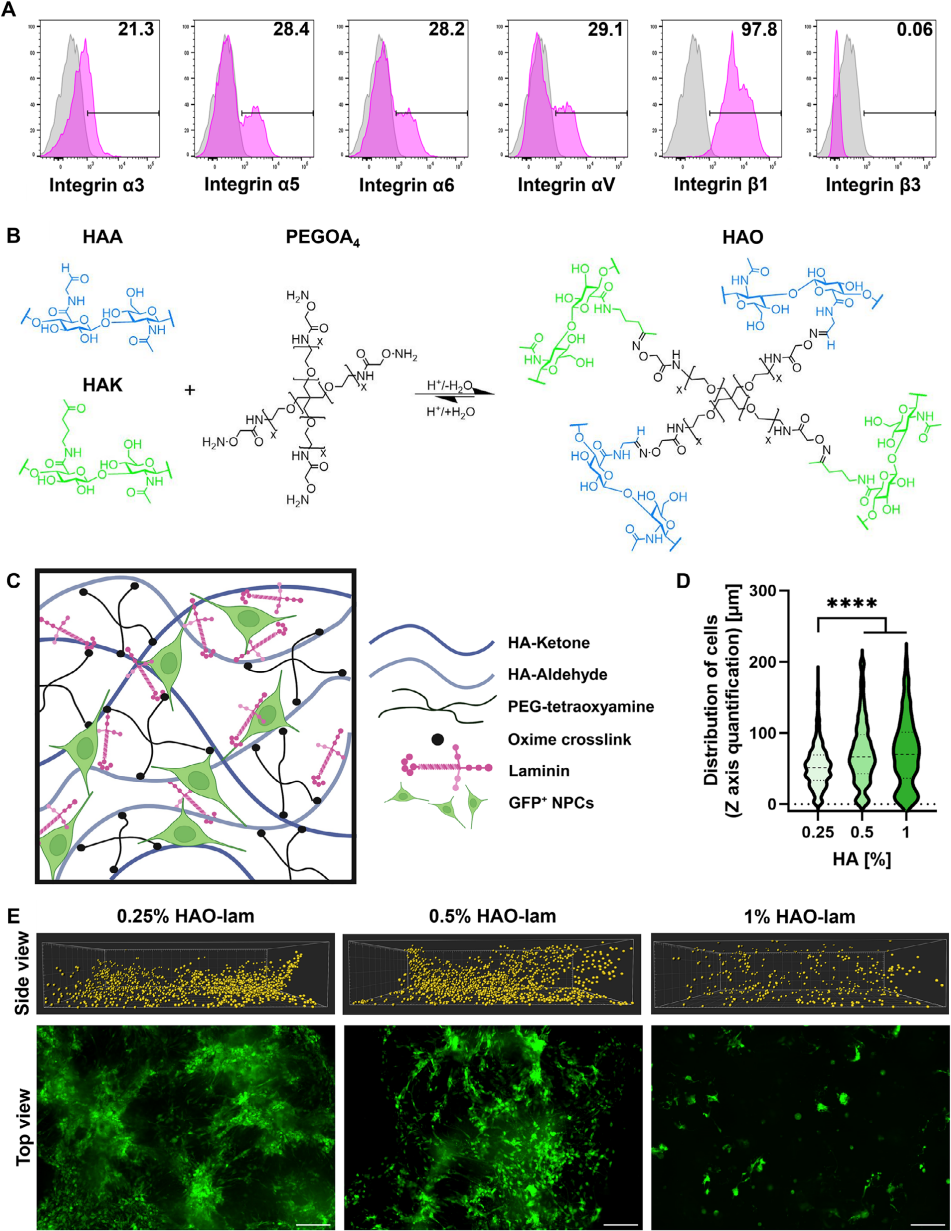
Vehicle design for the delivery of neural progenitor cells (NPCs). (A) Flow analysis of the expression of integrin receptors on the surface of NPCs. Magenta histograms indicate the positive population for each marker; integrin expression percentages are indicated in the upper right corner, while grey histograms indicate isotype controls. (B) Schematic of the reaction between hyaluronan‐aldehyde (HAA) and hyaluronan‐ketone (HAK) with poly(ethylene glycol)‐tetraoxyamine (PEGOA_4_) to form the hyaluronan‐oxime (HAO) hydrogel. (C) Schematic illustrating the composition of the delivery vehicle designed for NPCs. (D) Quantification of encapsulated GFP^+^ NPCs distribution along the z‐axis in HAO‐lam gels with increasing concentrations of HA. Data are reported as violin plots, *n* = 4 independent experiments, one‐way ANOVA with Tukey's post‐hoc, ^****^ *p* <0.0001. (E) Representative images of encapsulated cells. Cells (green, top view) were fixed, and nuclei (yellow, side view) were stained with Hoechst. Scale bar = 100 µm.

For cell transplantation, we differentiated GFP^+^ iPSCs into NPCs and subsequently toward a neuronal lineage in vitro. Differentiation was evaluated using immunocytochemistry (ICC) and qPCR for expression of neural, neuronal, and synaptic markers (Figure S1), which indicate neuronal commitment. We then designed a hydrogel to support cell survival and neuronal differentiation in vitro and in vivo, to leverage the impact of mechanical and biochemical cues on cell fate. We analyzed the expression levels of various cell surface receptors and found that integrins α3, α5, α6, αV, and β1 are all expressed by NPCs (Figure 2A). Integrin β1 (ITGβ1), also known as CD29, can interact with multiple α subunits to bind extracellular matrix (ECM) proteins: the combinations α_3_β_1_ and α_6_β_1_ specifically bind sequences on laminin [44, 45], which enhances NPC adhesion, migration, neurite extension, and neuronal differentiation [45, 46], making it compelling for incorporation into our cell‐delivery hydrogel.

### 2.2 Design of a Hyaluronic‐Acid (HA)‐Based Hydrogel as a Cell Delivery Vehicle

Hyaluronic acid (HA) is the main component of the brain's ECM; it can be easily modified and is known to affect cell behavior by interacting with cell‐surface receptors [19]. Ketone‐ and aldehyde‐modified HA were crosslinked using poly(ethylene glycol)‐tetraoxyamine (PEGOA_4_) to form HA‐oxime (HAO) hydrogels (Figure 2B). We added laminin prior to crosslinking, thereby forming HAO‐lam, to support cell survival (Figure 2C schematic). Laminin concentration impacted cell morphology (Figure S2A), number (Figure S2B), and distribution (Figure S2C). HAO‐lam gels supplemented with 2 mg/mL laminin had more cells extending neurites than those with 4 mg/mL laminin, which had no cells with neurites, potentially due to the greater stiffness of the gel: the stiffness of HAO‐lam with 2 mg/mL laminin was 0.40 ± 0.09 kPa whereas that of 4 mg/mL laminin was 1.69 ± 0.59 kPa. Interestingly, HA content also impacted cell number (Figure S3) and distribution (Figure 2D,E): 0.25% w/v HA in HAO‐lam gel had an uneven distribution of cells, with many at the bottom of the wells, likely because it was too soft; 0.5% w/v HA was optimal for cell survival and distribution; and 1% w/v HA resulted in reduced cell survival and neurite length, likely because it was too stiff. Indeed, the stiffness of 0.25% w/v HAO gel was too low to measure, yet that of 0.5% was 0.43 ± 0.05 kPa, and that of 1% was 4.89 ± 1.22 kPa. Therefore, the 0.5% HA and 2 mg/mL laminin HAO‐lam hydrogel was used in further experiments.

**FIGURE 3:**
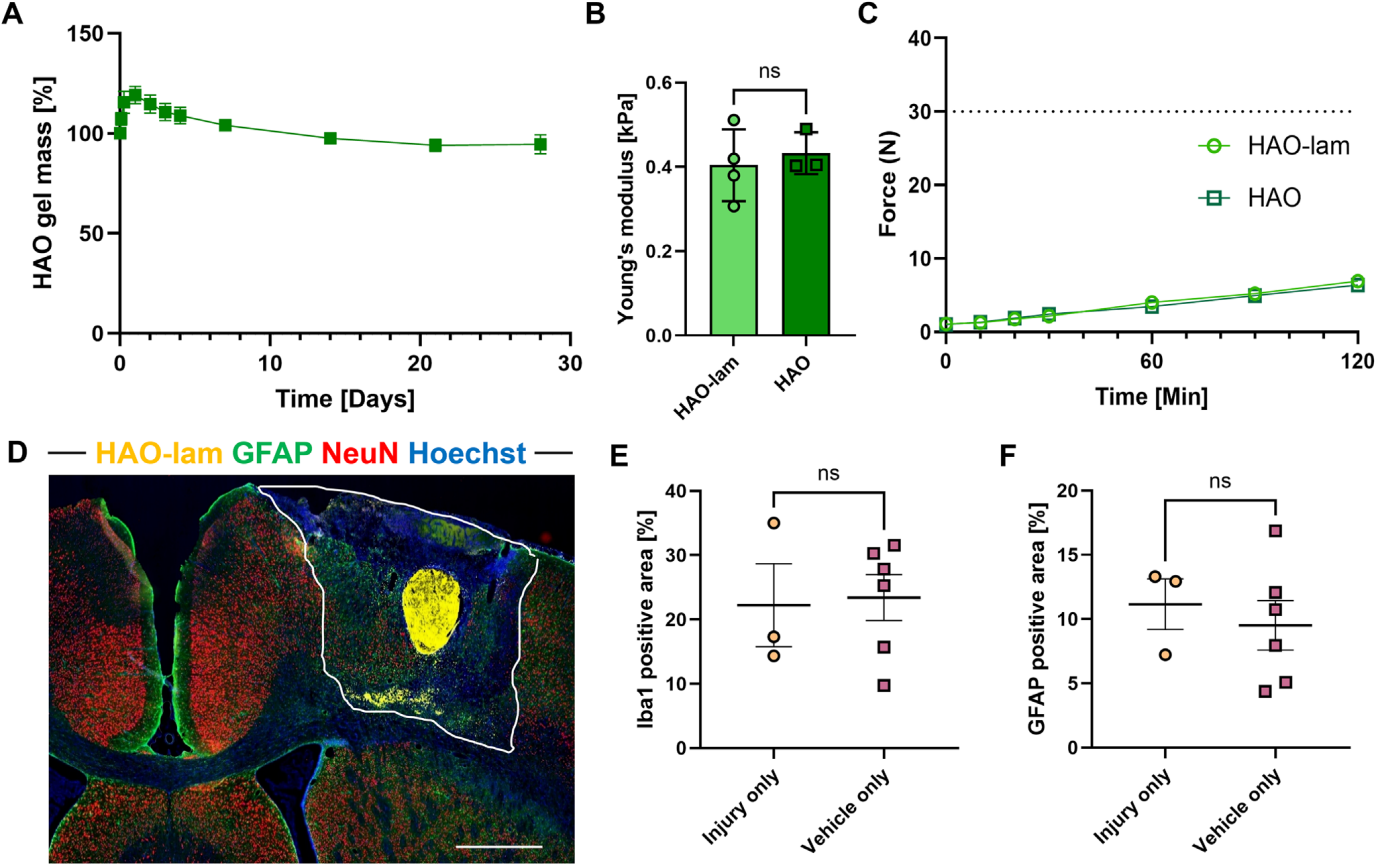
Characterization of HAO‐lam as a delivery vehicle for NPC transplantation. (A) HAO gel stability in vitro. (B) Young's modulus of HAO and HAO‐lam gels. (C) Injectability of HAO and HAO‐lam gels over time. In graphs A‐C, data are reported as mean ± SD, *n* = 3‐4 independent experiments. (D) HAO‐lam hydrogel (yellow) within the stroke lesion 48 h after injection. White solid line indicates lesion border according to lack of NeuN‐positive cells. Scale bar = 1000 µm. (E+F) Quantification of (E) Iba1^+^ microglia and (F) GFAP^+^ astrocytes in the lesion area, 48 h following hydrogel administration. *n* = 3‐6 animals per group; each dot represents one animal, and p‐values were determined by unpaired parametric t‐test. Data are reported as mean ± SEM. ns = not significant.

HAO hydrogels showed initial swelling of up to 19 ± 4.5% and remained stable for at least 28 days in PBS at physiological temperature and pH (Figure 3A). The incorporation of laminin (2 mg/mL) in the HAO hydrogels neither affected the Young's modulus of the gel (Figure 3B, 0.43 ± 0.05 kPa for HAO vs. 0.40 ± 0.09 kPa for HAO‐lam, as measured 24 h after gelation), nor the injectability through a fine (26 G) needle for at least 2 h (Figure 3C). Furthermore, both HAO and HAO‐lam exhibited rapid gelation as confirmed by rheology, with G′ > G″ as the accepted rheological definition of a gel (Figure S4). HAO and HAO‐lam were less stiff than the reported brain stiffness and therefore less likely to provoke a local inflammatory response, as previously established [45]; for example, bovine gray matter was reported to be ∼1.4 ± 0.3 kPa, with variations according to age and specific location [47]. Fluorescently labeled HAO‐lam was injected into the cavity of stroke‐injured rats 7 days post injury (DPI) to test its feasibility for later use as a cell delivery vehicle. The hydrogel remained within the lesion (Figure 3D) and did not elicit an additional inflammatory response, as shown (Figure S5) and quantified by IHC for microglia and reactive astrocytes (Figure 3E,F).

### 2.3 HAO‐Lam Successfully Supports NPC Survival and Differentiation In Vitro

**FIGURE 4:**
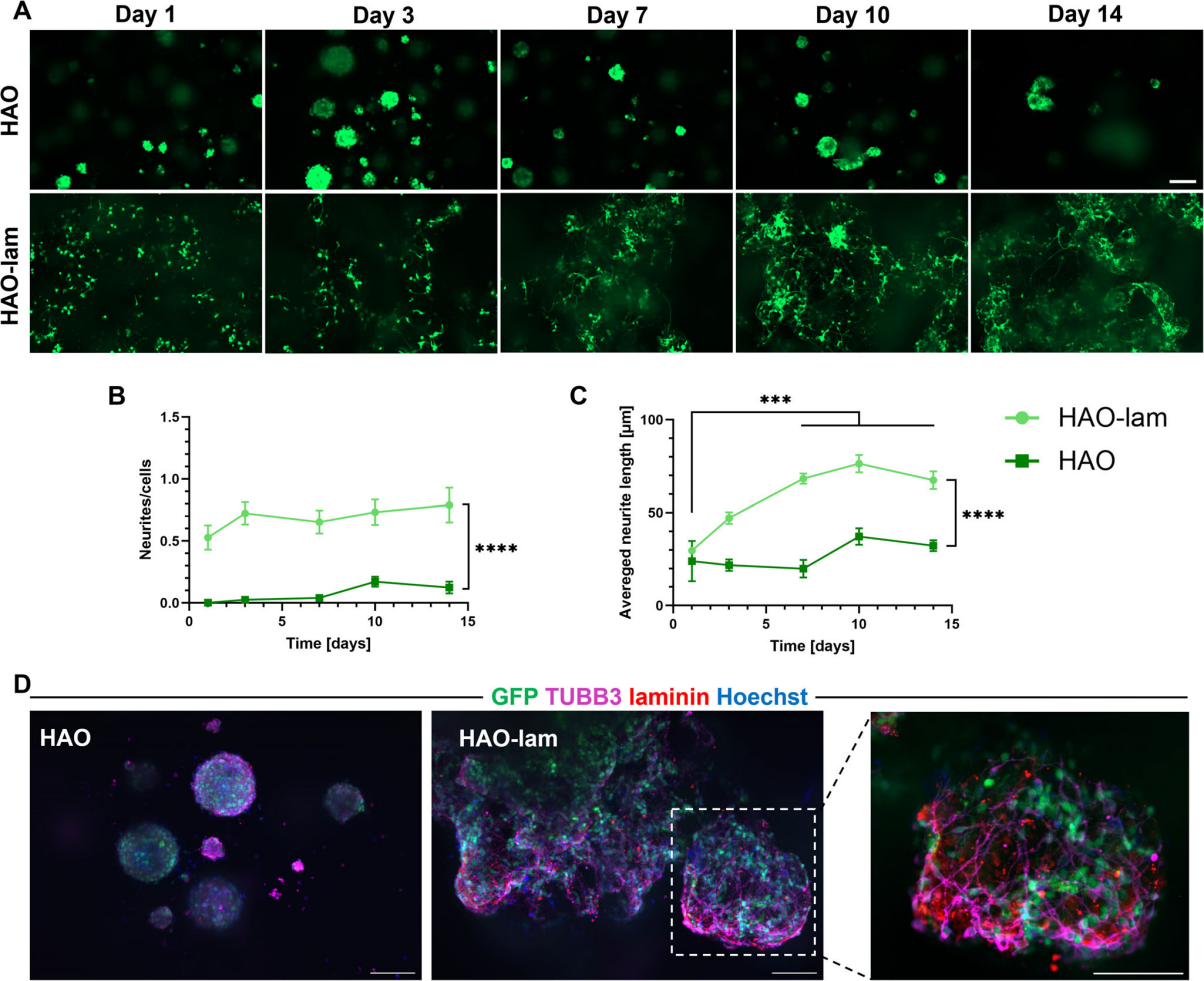
HAO‐lam supports survival and enhances commitment of NPCs into the neuronal lineage in vitro. (A) Representative images of cells encapsulated in HAO and HAO‐lam gels at 1, 3, 7, 10, and 14 days, in vitro. Scale bar = 100 µm. Quantification of (B) numbers and (C) length of neural projections in HAO and HAO‐lam gels over time. *n* = 4 independent experiments, data are presented as mean ± SEM, two‐way ANOVA with Šidák post hoc, ^***^ *p* <0.001, ^****^ *p* <0.0001. (D) Representative images of encapsulated cells in HAO and HAO‐lam at 7 d post‐encapsulation. TUBB3 staining (magenta) was used to confirm neuronal lineage. Scale bar = 100 µm.

NPCs remained clustered in HAO, whereas in HAO‐lam they interacted to form interconnected networks throughout the gel over 14 d in vitro (Figure 4A). Quantitative analysis confirmed the critical role of biochemical cues, such as laminin, in enhancing cell spreading and neurite outgrowth, with a significant increase in both neurite number (Figure 4B) and length (Figure 4C). Furthermore, after 7 d of culture, NPCs within the HAO‐lam hydrogels showed elevated expression levels of the early neuronal marker, β3‐tubulin (TUBB3), with neurites co‐staining with laminin fibers within the gel (Figure 4D).

We evaluated NPC survival and differentiation following fine‐needle injection in vitro. Pre‐injection viability was 97.7 ± 2.4% while post‐injection viability decreased to 84.8 ± 1.0% (in media) and 85.9 ± 1.3% (in HAO‐lam), indicating high survival with only a modest hydrogel effect (Figure S6A). The reduced viability likely reflects mechanical disruption during ejection, as cells experience extensional and shear forces while flowing through the needle [48]. Injected cells maintained in culture for one week expressed the early neuronal marker TUBB3, suggesting neuronal lineage commitment (Figure S6B).

### 2.4 Co‐Delivery of ChASE37‐AR and NPCs to the Stroke‐Injured Brain Enable Long‐Term Bioactivity Without Affecting Either Immune Response or Lesion Volume

To deliver ChASE37‐AR into the stroke‐injured brain, we used a crosslinked methylcellulose (MC)‐based hydrogel (XMC) as the delivery vehicle [41, 49]. As MC is an inverse thermogelling polymer, it naturally forms physical crosslinks. To create a chemically crosslinked hydrogel, we modified MC with thiol groups and reacted with PEG‐bismaleimide (PEG‐MI_2_) in a Michael‐type addition (Figure S7). ChASE37 (and ChASE) degrade hyaluronic acid, thus necessitating a different hydrogel for delivery: for example, ChASE37 (and hyaluronidase) degraded HAO within 4 days (Figure S8A) whereas only cellulase (and not ChASE37) degraded XMC within a week (Figure S8B). Notably, XMC cannot support NPC survival and differentiation (data not shown), necessitating the use of HAO‐lam as a cell delivery vehicle. XMC showed an initial swelling of up to 17 ± 2.7% and remained stable for at least a month in PBS at physiological temperature and pH (Figure S8C).

**FIGURE 5:**
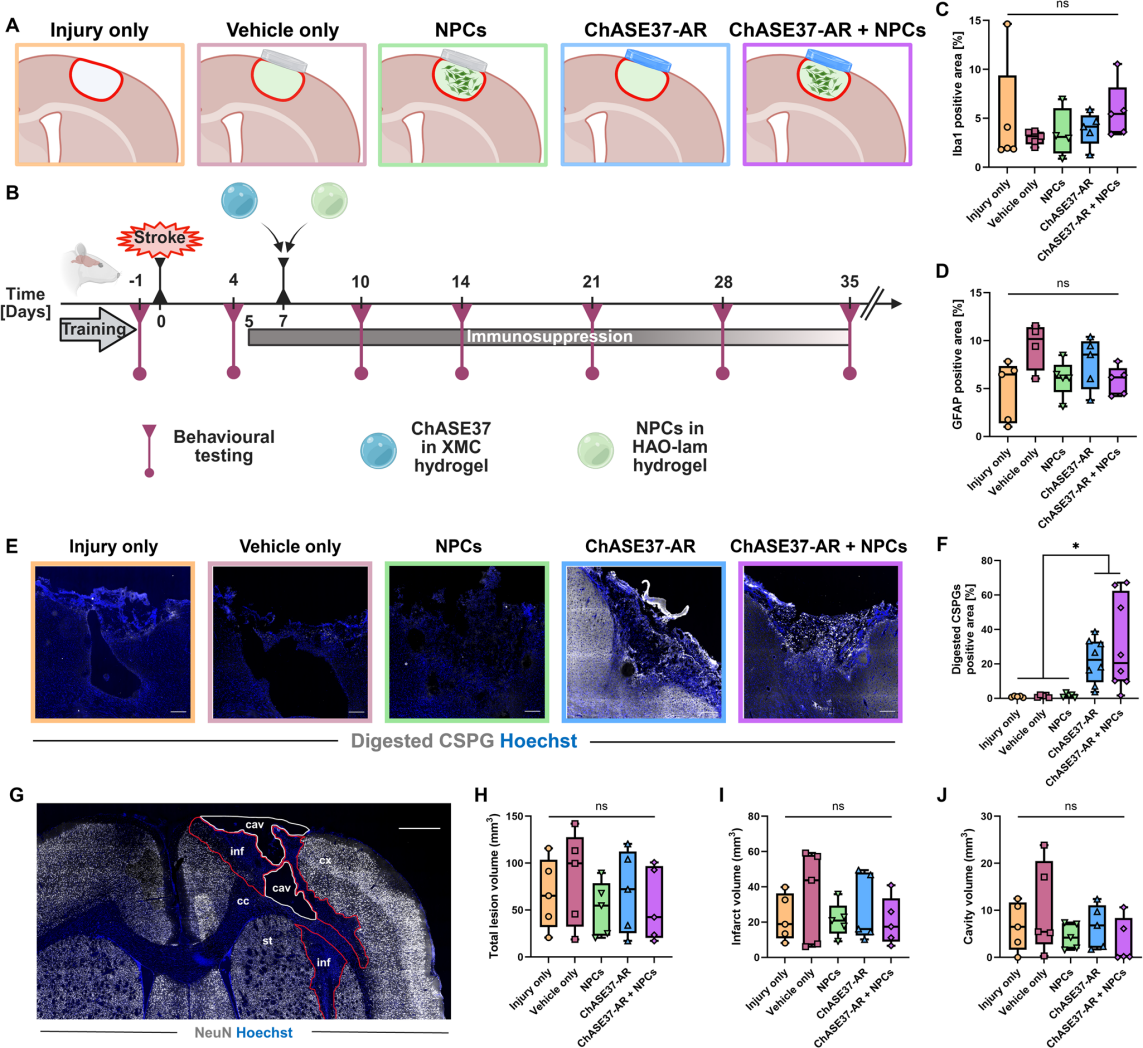
Co‐delivery of ChASE37‐AR with NPCs exhibits long‐term bioactivity without affecting either the immune response or lesion volume in the stroke‐injured rat brain at 35 DPI. (A) Schematics of treatment groups. Injury only: saline in the lesion and epicortically; Vehicle Only: HAO‐lam in the lesion and XMC epicortically; NPCs: NPCs encapsulated in HAO‐lam in the lesion and XMC epicortically; ChASE37‐AR: HAO‐lam in the lesion and ChASE37‐AR in XMC epicortically; ChASE37‐AR + NPCs: NPCs encapsulated in HAO‐lam in the lesion and ChASE37‐AR in XMC epicortically. (B) Study timeline. Quantification of (C) Iba1^+^ microglia and (D) GFAP^+^ astrocytes in the lesion. *n* = 5 animals per group. (E) Representative images of digested CSPGs immunostained with C4S (grey) and counterstained with Hoechst for nuclei (blue) in the stroke lesion areas in the different treatment groups. Scale bar = 100 µm. (F) Quantification of digested CSPGs immunostained with C4S, *n* = 5‐8 animals per group. (G) Representative image of the stroke lesion area. Lesion volume is defined as the sum of infarct (Hoechst^+^/NeuN^−^ tissue) and cavity (Hoechst^−^/NeuN^−^ tissue). Scale bar = 1000 µm; abbreviations: cav = cavity, cc = corpus callosum, cx = cortex, inf = infarct, st = striatum. Quantification of (H) total lesion, (I) infarct, and (J) cavity volumes. *n* = 5 animals per group. Data are presented using a min‐to‐max box plot. Each dot represents one animal, and p‐values were determined by one‐way ANOVA with Tukey's post‐hoc; ns = not significant, ^*^ *p* <0.05.

We investigated the co‐delivery of NPCs (50 000 cells/animal) with ChASE37‐AR in the rat endothelin‐1 stroke injury model to systematically assess the contribution of each component, as well as their combined therapeutic potential (Figure 5A for experimental design, Figure 5B for timeline). Following stroke, animals exhibited a transient decrease in body weight, with an average loss of 2.8% and a maximal loss of 12.8% from baseline measured at one‐week post‐injury (Figure S9A); however, body weight steadily increased thereafter across all treatment groups, approaching levels observed in healthy controls (Figure S9B). Since the aggregate scar thickens and matures over the first two weeks post‐injury in pre‐clinical models [30], we administered treatment at 7 days post injury (DPI), corresponding to the subacute phase in humans [50]. This timing allowed hydrogel delivery into an established cavity without increasing intracranial pressure [51], which coincided with waning inflammation [52], thereby favoring transplanted cell survival. Moreover, at 7 DPI, the maturing aggregate scar [30] was targeted, which is ideal for ChASE37 activity.

When examining animals from the various treatment groups, there were no significant differences in either Iba1^+^ microglia (Figure 5C) or GFAP^+^ astrocytes (Figure 5D), as evaluated by immunostaining at 4 weeks after treatment (35 DPI), indicating no adverse tissue reaction, beyond the injury itself, to any of the treatments or controls. ChASE37‐AR, whether delivered alone or with cells, degraded chondroitin sulfate proteoglycans (CSPGs) formed after stroke injury (Figure 5E). As expected, no CSPG degradation was observed in the other groups without ChASE37‐AR, and thus a significant difference in CSPG degradation was observed (Figure 5F). This demonstrates prolonged enzymatic activity of ChASE37 after a single injection, which we attribute to its extended bioavailability and slow release.

As a proxy for tissue regeneration across treatment groups, we quantified lesion volumes comprised of the infarct, which was characterized by the absence of neurons, and of the cavity, characterized by the absence of cells (Figure 5G). Although no statistically significant differences were observed, similar to previous studies [40, 49, 53], the NPCs groups (i.e., NPCs alone and ChASE37‐AR + NPCs) consistently showed lower mean values of total lesion (Figure 5H), infarct (Figure 5I), and cavity volumes (Figure 5J), suggesting either or both tissue sparing and regeneration associated with the co‐delivery treatment.

### 2.5 Co‐Delivery of ChASE37‐AR and NPCs Improves Behavioral Outcomes in the Stroke‐Injured Brain

**FIGURE 6:**
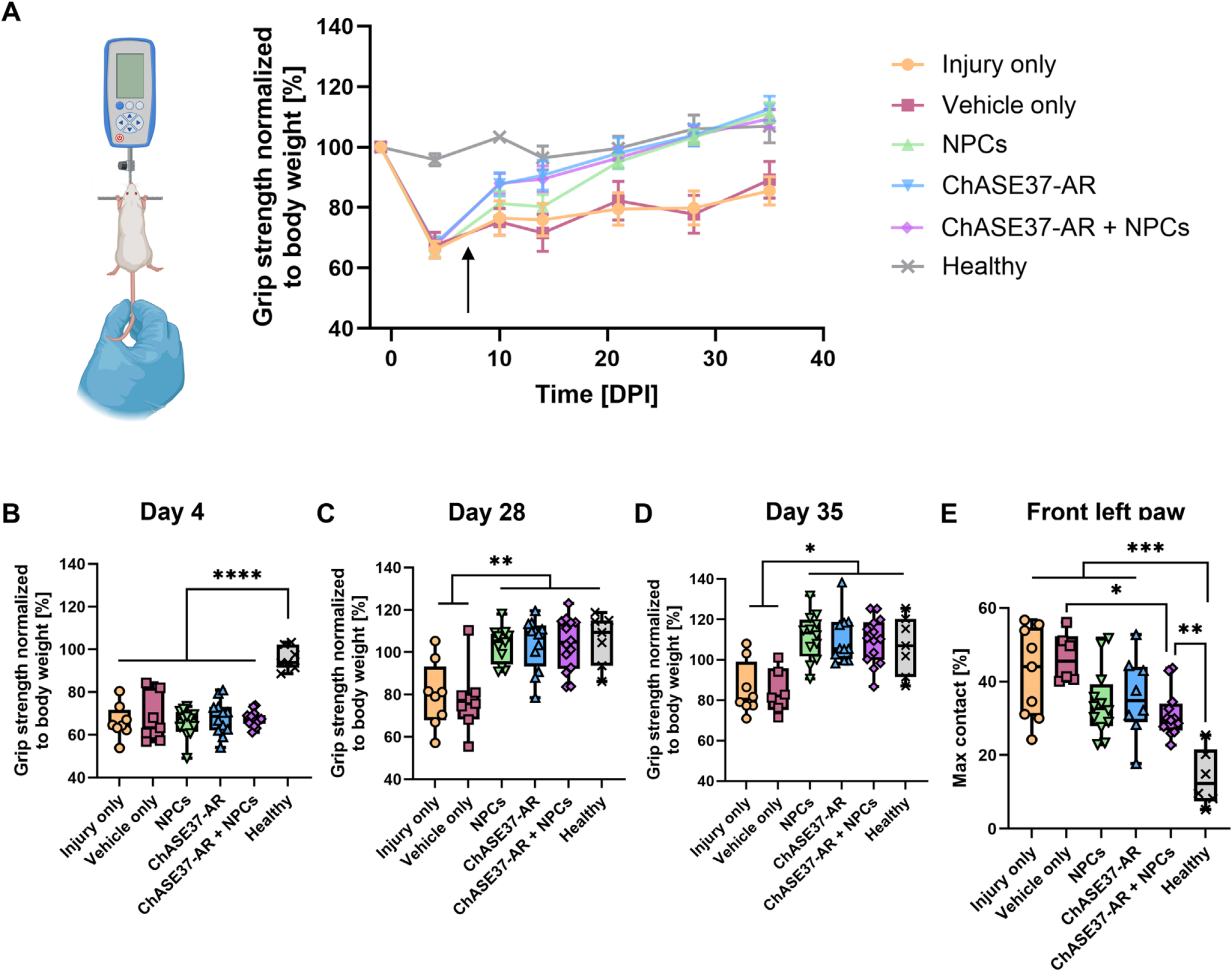
ChASE37‐AR, NPCs, and co‐delivery enhance recovery in an ET‐1 stroke injury model in rats. (A) Schematic of the grip strength assay, and grip strength results normalized to body weight over the course of the study (n = 7–14 animals per group, data are presented as mean ± SEM, a two‐way ANOVA with Šidák post‐hoc was used). Black arrow at 7 DPI indicates treatment injection. Individual grip strength (normalized to body weight) values at days (B) 4, (C) 28, and (D) 35 to better show the spread in the data and significant differences (n = 7–14 animals per group, one‐way ANOVA with Tukey's post‐hoc was used). (E) Percentage of maximal contact area of the left front paw, as measured by the CatWalk assay at 14 DPI. *n* = 6–12 animals per group, one‐way ANOVA with Tukey's post‐hoc. In graphs B‐E, data are presented using a min‐to‐max box plot. Each dot represents one animal. ^*^ *p* <0.05, ^**^ *p* <0.01, ^***^ *p* <0.001, ^****^ *p* <0.0001.

We measured muscle strength as a proxy for stroke‐related deficits and functional recovery using the grip strength assay (Figure 6A): at 4 DPI, all injured animals showed an average deficit of 34 ± 8% normalized to their individual, pre‐stroke baseline that was set at 100% (Figure 6B). After being treated at 7 DPI, we observed a steady increase in grip strength across the 3 treated groups, ChASE37‐AR, NPCs, and ChASE37‐AR + NPCs, as well as healthy controls. Compared to control groups of injury only and vehicle only, the treated groups showed improved recovery starting at 10 DPI with significantly greater muscle strength, ultimately reaching similar levels to those of healthy controls at 28 (Figure 6C) and 35 (Figure 6D) DPI.

To assess gait changes, we used the CatWalk system and observed few differences between the groups (Figure S10A–E); however, when we evaluated maximal contact area of the front left paw (i.e. contralateral side to the stroke injury) at 14 DPI, we observed a significant difference between the ChASE37‐AR + NPCs co‐treatment group vs. vehicle control, reflecting enhanced functional recovery (Figure 6E). This is consistent with our stroke model, which mostly targets the contralateral forelimb.

### 2.6 Co‐Delivery With ChASE37‐AR Enables the Survival and Differentiation of Transplanted NPCs in the Stroke‐Injured Rat Brain

**FIGURE 7:**
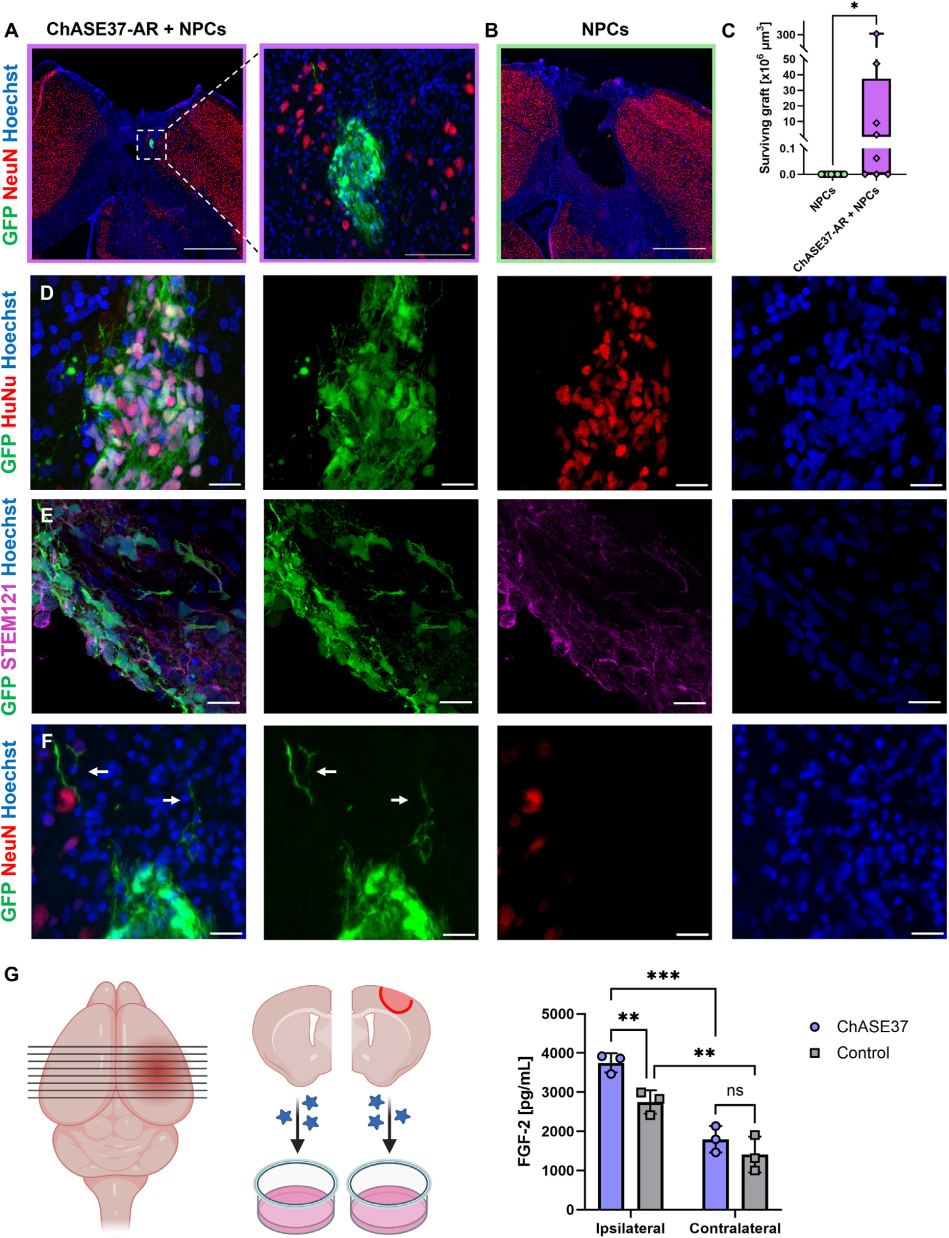
Co‐delivery with ChASE37‐AR enables survival of human NPCs in the stroke‐injured rat brain. (A) Representative image of the ChASE37‐AR + NPCs treatment showing surviving cells in the stroke lesion; and magnification of the graft, showing the GFP^+^ cells. Scale bars = 1000 and 100 µm, respectively. (B) Representative image of the NPCs treatment showing no evidence of surviving cells. Scale bar = 1000 µm. (C) Quantification of surviving graft volumes in each of the transplanted groups. *n* = 8 animals per group; data are presented using a min‐to‐max box plot. Each dot represents one animal, and p‐values were determined by an unpaired nonparametric t‐test, ^*^ *p* <0.05. GFP^+^ cells are co‐labeled with (D) the human nuclei marker, HuNu; and (E) the human cytoplasmic marker, STEM121, confirming their human origin. (F) GFP^+^ projections are observed migrating into host tissue (labeled by white arrows). Panels D‐F: scale bar = 20 µm. (G) Schematic of the experimental setup and the quantification of FGF‐2 in the conditioned media of ipsilateral vs. contralateral stroke‐injured brain sections after incubation with either ChASE37 (1 U/mL in aCSF) or aCSF alone (controls). *n* = 3 animals; each data point represents one animal; p‐values were determined by a two‐way ANOVA with Šidák post‐hoc analysis; ^**^ *p* <0.01, ^***^ *p* <0.001.

We found transplanted cells 4 weeks after co‐delivery of ChASE37‐AR + NPCs (Figure 7A), but not in the NPCs alone group (Figure 7B). Interestingly, we observed cells in 5 out of 8 animals in the co‐delivery group vs. none in the NPCs alone group (Figure 7C). We confirmed that the GFP^+^ cells originated from the transplanted human NPCs by co‐staining with human‐specific antibodies, HuNu (Figure 7D, nuclear marker) and STEM121 (Figure 7E, cytoplasmic marker). Fascinatingly, long extensions emanating from the transplanted human NPCs into the host tissue were observed (Figure 7F, highlighted by white arrows), and their expression of the early neuronal marker TUBB3 was confirmed (Figure S11A‐B). Location within the lesion is shown in Figure S11A. Further immunohistochemical analysis revealed dispersed HA and laminin around the graft (Figure S12), rather than a persistent bulky hydrogel structure. Given the presence of endogenous HA and laminin in brain tissue, partial HAO‐lam degradation is not expected to adversely affect host tissue. Moreover, in vitro exposure to ChASE37‐mediated degradation products did not affect NPC viability (Figure S13).

To better understand the mechanism underlying cell survival following ChASE37‐AR delivery, we incubated freshly isolated ipsilateral vs. contralateral hemisphere brain slices from stroke‐injured rats with either ChASE37 in aCSF or aCSF alone (controls), and then analyzed the conditioned media (CM, Figure 7G). The addition of ChASE37 to the ipsilateral hemispheres, where CSPG expression is increased following stroke, significantly increased the release of fibroblast growth factor 2 (FGF‐2) compared to controls (3,749 ± 248 vs 2,746 ± 300 pg/mL, respectively). In contrast, FGF‐2 levels in the CM were significantly lower in the contralateral hemispheres, with no differences between ChASE37‐treated and control groups (1,798 ± 336 vs 1,406 ± 460 pg/mL, respectively).

**FIGURE 8:**
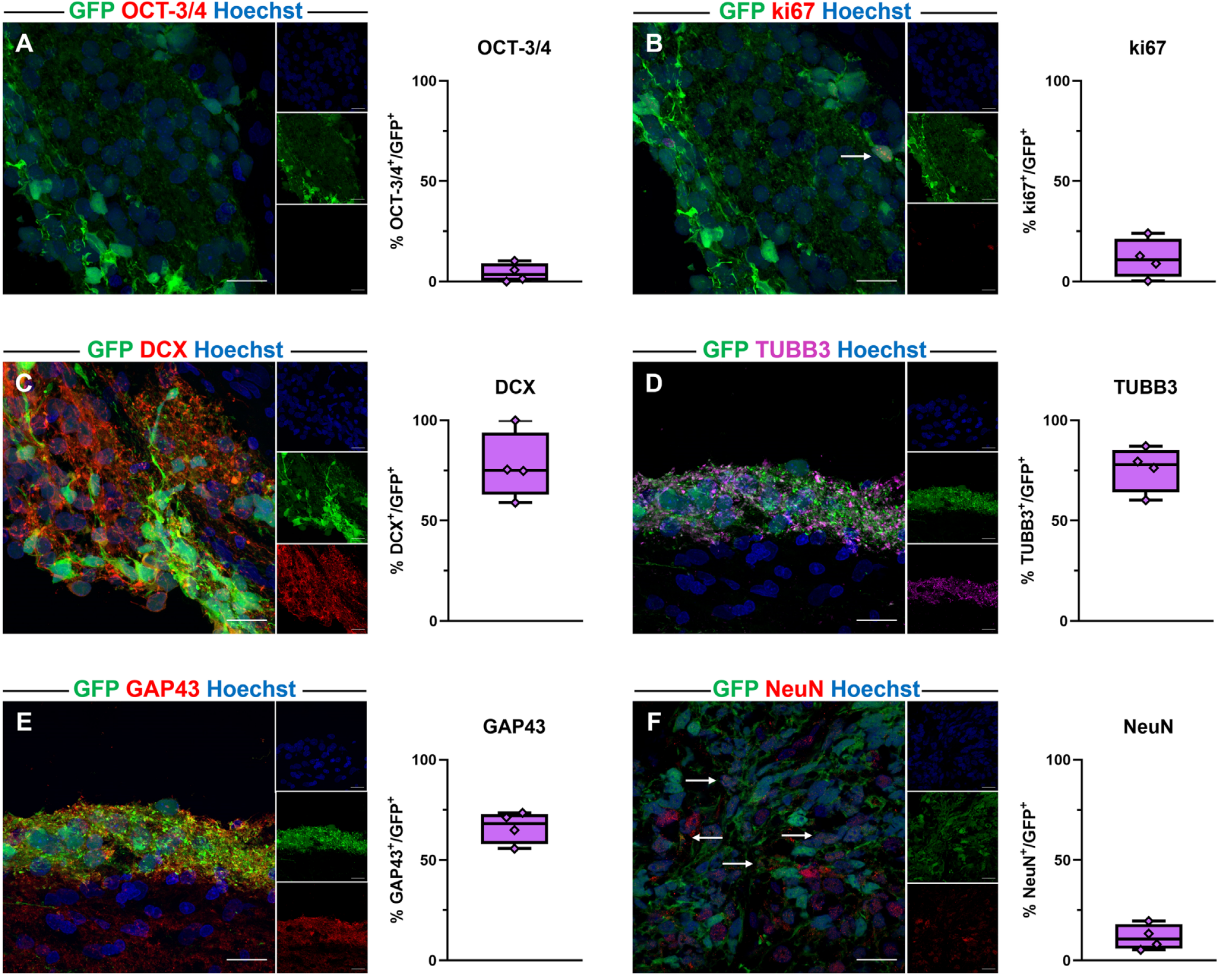
Human NPCs in the co‐delivery group express high levels of neuronal markers 4 weeks post‐transplantation. Representative merged images (left) display Hoechst, GFP, and the appropriate marker with individual channels (right), with quantification of (A) OCT3/4, (B) Ki67, (C) DCX, (D) TUBB3, (E) GAP43, and (F) NeuN. Scale bar = 20 µm. *n* = 4 animals; data are presented using a min‐to‐max box plot. Each dot represents one animal.

The surviving transplanted NPCs, when co‐delivered with ChASE37‐AR, had minimal expression of the pluripotency marker, Oct3/4 (4.3 ± 4.6%, Figure 8A) and low levels of the proliferation marker Ki67 (11.5 ± 9.8%, Figure 8B), suggesting minimal risk of undifferentiated cell proliferation and formation of teratomas. In contrast, the surviving NPCs exhibited high expression levels of early neuronal markers: DCX for migrating neuroblasts at 77.3 ± 16.9% (Figure 8C); TUBB3 for immature neurons at 75.8 ± 11.3% (Figure 8D); GAP43 for cytoplasmic proteins involved in neurite outgrowth and plasticity at 66.4 ± 8.0% (Figure 8E); as well as GFAP, the astrocytic marker, at 49.9 ± 17.5% (Figure S14), suggesting some astrocytic differentiation. Additionally, a small subset of cells expressed NeuN at 11.6 ± 6.3% (Figure 8F), suggesting partial neuronal maturation within the surviving transplanted population. Interestingly, in a parallel in vitro study, a subset of NPCs maintained for quality control throughout the study spontaneously differentiated into a neuronal‐committed state as characterized by extended neurites and the expression of early neuronal markers TUBB3 and DCX in addition to the neuronal marker NeuN (Figure S15).

## 3 Discussion

In this study, we examined the combined effect of sustained affinity release of ChASE37‐AR and human iPSC‐derived NPCs on tissue regeneration and functional recovery in the endothelin‐1 rat stroke model.

Cell transplantation has been investigated in a diversity of disorders and injuries both pre‐clinically and clinically. It is particularly attractive in the context of stroke because exogenous cells have the potential to both replace lost tissue and secrete regenerative factors to support endogenous host tissue regeneration [54, 55]. Numerous factors influence the success of cell therapy, including the source and maturity of the cells, cell dose, and delivery route, as outlined in recent reviews [9, 55]. However, one of the primary barriers to clinical translation remains the poor long‐term survival of transplanted cells [9]. To address this, we co‐delivered NPCs in a supportive cell‐delivery vehicle with ChASE37‐AR. Notably, ChASE37‐AR could not be delivered in the same hydrogel with NPCs as HAO is rapidly degraded by ChASE37. In the absence of ChASE37 and hyaluronidase, HAO is stable at physiological temperatures and pH; however, hyaluronidase is upregulated in rodents after stroke [56, 57], indicating that HAO‐lam will be degraded.

Consistently, we observed scattered HA and laminin surrounding the graft at 4 weeks post‐transplantation, with no evidence of a persistent bulky hydrogel structure, suggesting progressive degradation of the implanted material. While it is difficult to distinguish between exogenous and endogenous HA and laminin, the small volume (4 µL) of HAO‐lam injected did not impede regeneration, is permissive to cellular infiltration, and thus should support gradual tissue remodeling.

ChASE37‐AR mediated survival of NPCs in the co‐delivery group, which is an exciting discovery. Although we did not examine cell survival at early time points, we hypothesize that cell loss occurred soon after transplantation, likely due to the pro‐inflammatory and inhibitory microenvironment, based on both our studies and those of others [58]. The improved NPC survival observed with ChASE37‐AR delivery correlates with the prolonged bioactivity of ChASE37, as demonstrated by CSPG degradation at 4 weeks after injection (5 weeks after injury). Degradation of CSPGs attenuates its inhibitory effect on axon regeneration, making the microenvironment more amenable to NPC survival. Moreover, the neuroprotective effect may be attributed to the release of chondroitin sulfate‐bound growth factors due to CSPG degradation [38]. Several growth factors, such as BDNF and FGF‐2, are known to bind CS‐GAGs with high affinity [24], and their release can enhance tissue regeneration. Our ex vivo investigation showed significantly more FGF‐2 released from stroke‐injured, ipsilateral brains treated with ChASE37 than aCSF (vehicle control). FGF‐2 levels in conditioned media from contralateral brains were significantly lower than those from the corresponding ipsilateral tissues, which is consistent with previous reports that show increased FGF‐2 expression following ischemic stroke [59, 60]. Furthermore, ChASE37 treatment did not increase FGF‐2 release from the contralateral tissue, likely reflecting reduced CSPGs in the uninjured tissue compared with the CSPG‐rich injured hemisphere, and hence less matrix‐sequestered FGF‐2 available for enzymatic release. Since FGF‐2 has been shown to enhance progenitor cell differentiation, neuronal survival, and neurogenesis [61, 62], our hypothesis that ChASE37‐AR supports transplant cell survival is further substantiated. We appreciate that additional in‐depth molecular and cellular investigations will be required to fully elucidate the underlying regenerative mechanisms associated with the therapeutic efficacy observed with the combined delivery of NPCs and ChASE37‐AR.

In addition to greater survival with ChASE37‐AR co‐delivery, we observed little NPC proliferation, which differs from other reports where transplanted NPCs proliferated or differentiated mainly into astrocytic phenotypes [53, 63]. Although OCT3/4 is robustly downregulated during neural differentiation in vitro, low‐level expression can persist in early neural progenitors or be transiently re‐expressed in vivo in response to stress cues [64, 65, 66]. This suggests that the minimal OCT3/4 signal observed in surviving grafts likely reflects residual or microenvironment‐driven expression rather than maintenance of pluripotency. This is supported by the strong expression of early neuronal markers, such as β3‐tubulin and doublecortin, and the early astrocytic marker GFAP. GFAP is expressed by iPSC‐derived NPCs [67], yet its expression decreases during neuronal commitment, as we have seen in spontaneous differentiation in vitro. The higher expression levels seen in vivo likely reflect the effects of the post‐stroke microenvironment on the cells, which may increase differentiation towards an astroglial fate. Further characterization would be required to identify these cells as astrocytes [67]. Interestingly, we also observed some expression of the mature neuronal marker, NeuN. This may be attributed to the composition of the HAO‐lam hydrogel used for NPC delivery, as both HA and laminin have been reported to support neuronal differentiation [45, 46]. The extensions from exogenous NPCs into the host tissue suggest integration into the endogenous tissue, which would be confirmed with a longer study, as it typically takes months for human cells to integrate into rodent brains [68, 69, 70].

The grip strength test, which objectively quantifies maximal forelimb force, serves as a measurement of the animal's muscular strength. It is commonly used in stroke models and correlates with motor function in human stroke patients, who typically exhibit a significant (∼50%) grip force reduction in the affected hand. While this improves considerably in the first few weeks following stroke, it plateaus within the first 12 months [71]. Interestingly, despite the lack of cell survival in the NPCs‐only group, functional recovery was observed. This may reflect a transient bystander effect, where cells secrete neurotrophic or anti‐inflammatory factors before being cleared away. This is consistent with previous cell transplantation studies [72, 73], as well as with our own preliminary in vitro results (not shown), where multiple factors are secreted by iPSC‐NPCs, including angiopoietin 2 and VEGF‐A. Notably, healthy and treated animals slightly reached muscle strength of over 100% of initial values at the end of the study. This can be attributed to maturation to young adult male rats, as others have reported an increase in muscle strength of young adult rodents as they age [74, 75, 76]. Notably, no direct correlations were identified between histological endpoints and functional outcomes across treatment groups, similar to observations in other studies [77, 78]. The comparable recovery in grip strength among the three treatment groups highlights the need for a more sensitive behavioral assay (e.g., kinematic analysis) in future studies.

While we had anticipated significant changes in motor function and gait abnormalities using the CatWalk XT assay, we observed minimal post‐injury deficits and recovery. A meta‐analysis of rodent CatWalk data by Timotius et al. concluded that smaller, site‐specific damage, such as our mild stroke model, would have less effect on the gait [79]. The ET‐1 model produces a lesion that targets the forelimb motor region of the cortex and the dorsolateral striatum. It is known to cause significant and long‐lasting deficits in forelimb reaching and asymmetry, and postural support, which may be insufficient to be visualized on the CatWalk XT assay [42].

ChASE has been investigated in pre‐clinical models of SCI for several decades using repeated injections [80] or mini osmotic pumps [37]; however, its short half‐life and the required local sustained delivery have limited progress. Lentiviral delivery may provide a solution but is non‐targeted and will likely last longer than required [81, 82]. Notwithstanding the limitations observed in CNS repair, native ChASE is approved for the treatment of lumbar disc herniation (LDH) in Japan, and phase 3 clinical trials were recently completed in the US to alleviate LDH‐related leg pain [83, 84]. We anticipate that ChASE37‐AR, which overcomes some of the key limitations of native ChASE and lentiviral delivery, will provide improved therapeutic benefit and will also advance to clinical trials.

Cell therapy for stroke is progressing, with various delivery routes under investigation. Intracerebral delivery of autologous mesenchymal stem cells in subacute stroke patients showed 12‐month survival in a small trial (UMIN000026130) [85] while intravenous delivery of allogeneic multipotent cells (MultiStem) in the phase 2/3 TREASURE trial (NCT02961504) showed safety, but no efficacy in acute stroke patients (18–36 h of ischemic stroke onset) [86]. These efforts underscore both the promise and the complexity of translating cell‐based therapies into clinical practice, as well as the necessity of designing better ways to ensure successful cell survival post transplantation. Together, the integration of iPSC‐derived NPCs capable of neuronal differentiation, a supportive hydrogel matrix, and sustained‐release ChASE37‐AR represents a promising strategy that not only enhances current therapeutic approaches for stroke but also advances the frontier of regenerative medicine for CNS repair.

## 4 Conclusions

We demonstrated the benefit of delivering human iPSC‐derived NPCs in the HAO‐laminin hydrogel with sustained release of thermostabilized ChASE37: NPCs survived and differentiated primarily toward a neuronal lineage in the stroke‐injured rat brain when delivered with ChASE37‐AR, but not when delivered alone. ChASE37 remained bioactive in vivo, effectively degrading inhibitory CSPGs within the lesion 4 weeks after a single application, which correlated with neurite projections observed in host tissue. All treatment groups—ChASE37‐AR, NPCs, and ChASE37‐AR + NPCs—showed functional recovery relative to control groups by grip strength, with additional locomotor function observed in the ChASE37‐AR + NPCs co‐delivery group by CatWalk. The thermostability of ChASE37‐AR, coupled with its sustained release from a biocompatible hydrogel, provides a promising strategy to modulate the hostile microenvironment post‐injury. ChASE37‐AR combined with NPCs in biocompatible scaffolds is promising for continued investigation in the context of stroke and other traumatic injuries, including spinal cord injury.

## 5 Methods

### 5.1 In Vitro Cell Culture and Differentiation

The Zan11 cell line, generously provided by the Zandstra lab at UBC, Canada, was initially derived from activated CD3^+^ T cells isolated from human umbilical cord blood and modified to stably express GFP [87]. Human induced pluripotent stem cells (iPSCs) were maintained on Corning Matrigel (Sigma–Aldrich) in mTeSR Plus media (STEMCELL Technologies), with daily media changes. These iPSCs were differentiated into mature neurons following established protocols [88, 89] with minor adjustments, as previously reported [40]. Briefly, 3 × 10^6^ cells were seeded into a 24‐well Aggrewell 800 plate (STEMCELL Technologies) to form embryoid bodies (EBs) in Essential 6 (E6) media (Thermo Fisher Scientific) containing 10 µM Rock inhibitor Y27342 (Selleck Chemicals) and 2 µM each of DAP: Dorsomorphin (Tocris), A83‐01 (Tocris), and PNU (2‐Phenoxybenzoic acid‐[(5‐methyl‐2‐furanyl)methylene]hydrazide, R&D). The E6+DAP medium was replaced daily. After one week, EBs were collected, plated on Matrigel in E6+DAP medium, and the medium was changed daily for two weeks. The cells were classified as neural progenitor cells (NPCs), which were then expanded and cryopreserved. The cells were dissociated using Accutase (Sigma–Aldrich) for 5–10 min, centrifuged, and replated on Matrigel at a 1:2 ratio in neural media (DMEM: F12 (Gibco), supplemented with 0.2% β‐mercaptoethanol (Thermo Fisher Scientific), 1% N2 (Wisent Bioproducts), 2% B27 (Gibco), and 0.05% BSA‐FV (7.5% solution, Thermo Fisher Scientific)). The media was changed every other day and supplemented with 20 ng/mL basic fibroblast growth factor, FGF‐2 (R&D). From passage 3 onwards, cells were cultured on poly‐L‐ornithine (PLO, Sigma–Aldrich)/laminin (R&D). Neuronal differentiation was induced by adding 0.1 µm Compound E (STEMCELL Technologies) to the neural media for 2 d, followed by 20 ng/mL BDNF and GDNF (Peprotech). Media was replaced every 3–4 d for 4 weeks to achieve full differentiation.

### 5.2 RNA Extraction and Quantification Using qPCR

Cells in the different differentiation phases were washed in phosphate‐buffered saline (PBS), lysed, and RNA was extracted using the Nucleospin RNA II Kit (Macherey–Nagel) kit, according to the manufacturer's instructions. Purified RNA was stored at −80°C until cDNA synthesis, where 250 ng of RNA was used in 20 µL reaction using the Superscript VILO cDNA synthesis kit (Invitrogen). qRT‐PCR reactions (10 µl) were performed on a QuantStudio 6 Flex Real‐Time PCR System (Thermofisher Scientific), using Advanced SYBR Green (Bio‐Rad Laboratories) and 1 µm of primers (listed in Table S2). GAPDH served as a reference gene, to which cycle threshold (Ct) values were normalized to (ΔCt), and then normalized to undifferentiated iPSCs (ΔΔCt). Fold changes were calculated and plotted.

### 5.3 Immunocytochemistry (ICC)

Cells in the different differentiation phases were washed twice in PBS, then fixed with 4% paraformaldehyde (PFA) for 12 min, followed by 3× PBS wash. Permeabilization using Triton X‐100 (0.3%) in PBS was done for 15 min at room temperature (RT), followed by 3× PBS wash. Cells were blocked for 1 h at RT using donkey serum (10%) and Triton X‐100 (0.1%) in PBS, then incubated overnight (ON) at 4°C with primary antibodies in the presence of donkey serum (1%) and Triton X‐100 (0.1%), according to the dilutions in Table S1. The next day, cells were washed 3× with Triton X‐100 (0.05%) in PBS, followed by 1 h incubation with the appropriate secondary antibodies at RT. After a PBS wash, cells were counterstained with Hoechst 33342 (Cell Signaling) for 10 min at RT, washed 3× in PBS, and mounted using Prolong Gold (Invitrogen). Images were taken on a Zeiss LSM 880 confocal microscope.

### 5.4 Integrin Expression Using Flow Cytometry

NPCs were dissociated with Accutase and washed in PBS. For each sample, 2 × 10^5^ cells were incubated with PE‐conjugated antibodies (1:200, BioLegend; antibodies are listed in Table S1) for 30 min on ice in FACS buffer (0.5% bovine serum albumin, BSA, and 0.05% sodium azide in PBS). Following a PBS wash, cells were labeled with 7‐AAD viability dye (1:50, BioLegend) to discriminate dead cells. Samples were analyzed using an LSR Fortessa flow cytometer and FlowJo v10.7 software (BD Biosciences).

### 5.5 Synthesis of Hyaluronic Acid‐Based Hydrogel Components

#### 5.5.1 Ketone‐Modified Hyaluronic Acid (HAK)

HAK was synthesized as previously reported with minor modifications [20, 21]. Briefly, 1.0 g of sodium hyaluronate (229 kDa, Lifecore) was dissolved in 100 mL of 2‐(N‐morpholino)ethanesulfonic acid monohydrate (MES) buffer (0.1 M, pH 6.6). Next, 2x molar equivalent of 4‐(4,6‐dimethoxy‐1,3,5‐triazin‐2‐yl)‐4‐methyl‐morpholinium chloride (DMTMM, TCI Chemicals) was added to activate the HA carboxylates for 30 min, followed by the dropwise addition of 1x molar equivalent of 3‐(2‐methyl‐1,3‐dioxolan‐2‐yl)propanamine (ketal, Key Organics) under stirring for 48 h at RT to form hyaluronic acid‐ketal. The solution was dialyzed first against 0.1 m NaCl for 24 h (12–14 kDa molecular weight cut‐off), followed by 0.1 m HCl for 2 h to deprotect the ketal, then against 0.025 m sodium phosphate monobasic for 24 h, and finally against distilled water for 24 h to form hyaluronic acid‐ketone (HAK). HAK was then lyophilized, and the degree of substitution was analyzed by ^1^H NMR in deuterium oxide and calculated as 43 ± 1%.

#### 5.5.2 Aldehyde‐Modified Hyaluronic Acid (HAA)

HAA was synthesized similarly, and as previously reported with minor modifications [20, 21]. Briefly, 1.0 g sodium hyaluronate (229 kDa) was dissolved in 100 mL of MES buffer (0.1 m, pH 5.5). Next, HA carboxylates were activated using 1x molar equivalent of DMTMM for 30 min, followed by the dropwise addition of 0.5x molar equivalent of amino acetaldehyde dimethyl acetal (Sigma–Aldrich) under stirring for 3 h at 60°C to form hyaluronic acid‐acetal. The solution was dialyzed first against 0.1 m NaCl for 24 h (12–14 kDa molecular weight cut‐off), followed by 0.2 m HCl for 48 h to deprotect the acetal, then against 0.025 m sodium phosphate monobasic for 24 h, and finally against distilled water for 24 h to form hyaluronic acid‐aldehyde (HAA). HAA was then lyophilized, and the degree of substitution was analyzed by ^1^H NMR in deuterium oxide and calculated as 52 ± 1%.

#### 5.5.3 Polyethylene Glycol Tetraoxyamine (PEGOA_4_)

Boc‐aminooxyacetic acid (0.32 g, AK Scientific) was dissolved in 5 mL of anhydrous dichloromethane at 0°C in an ice bath and under nitrogen for 15–30 min. N, N'‐diisopropylcarbodiimide (DIC, 0.45 mL) was added and reacted for 1 h to activate the carboxylic acid groups. Then, 4‐arm polyethylene glycol (PEG)‐amine (1.0 g, MW 5000, JemKem Technology) was added, followed by N, N‐diisopropylethylamine (DIPEA, 0.53 mL, Sigma–Aldrich), and the reaction was stirred for 48 h at RT. The solvent was removed by vacuum, and the crude product was stirred with distilled water and filtered to remove any by‐products. The filtrate was then dialyzed against 0.1 m NaCl for 24 h, followed by 0.2 m HCl for 48 h for Boc deprotection, and finally against distilled water for 48 h. PEG‐tetraoxyamine (PEGOA_4_) was then sterile filtered and lyophilized. ^1^H NMR was used to determine completion of the reaction.

#### 5.5.4 HAO Gel Synthesis

HAK and HAA were reconstituted separately at 15 mg/mL in PBS, combined at a 1:1 v:v ratio, and autoclaved. Unless mentioned otherwise, all HAO gels were assembled as 0.5 wt.% HA, 2 mg/mL laminin, and 70 mol% PEGOA_4_, with the remaining volume being PBS or cell suspension in complete growth media. Hydrogels were vortexed to reach homogeneity and immediately dispersed into wells or tubes, as appropriate.

### 5.6 Synthesis of Methylcellulose‐Based Hydrogel Components

#### 5.6.1 Methylcellulose Synthesis

Crosslinked methylcellulose (MC) hydrogels (XMC) were synthesized as previously described [90, 91]. Briefly, MC (2.0 g, 300 kg/mol, Shin‐Etsu Corp.) was dissolved in 200 mL of 1.5 m NaOH solution at 4°C. Bromoacetic acid (15 g, Sigma–Aldrich) was added, and the reaction was stoppered and stirred for 48 h at 4°C. NaH_2_PO_4_•H_2_O (2.8 g) and HCl (6 m) were added to neutralize the reaction. The crude mixture was dialyzed against 0.1 m NaCl for 24 h (12–14 kDa molecular weight cut‐off), followed by water for 48 h, before being lyophilized to yield sodium carboxymethyl methylcellulose (MC‐COONa). Yield = 1.8 g. Next, MC‐COONa (0.54 g) was dissolved in 200 mL of 0.1 m MES buffer (pH 5.5) at 4°C. DMTMM (2.1 g) was added, followed by 3,3'‐dithiobis(propanoic acid) 1,1'‐dihydrazide (DTP, 0.91 g) 45 min later. The reaction was stoppered and stirred for 72 h at 4°C. The crude was dialyzed against 0.1 m NaCl at 4°C followed by water (both corrected to pH 4 using 6 m HCl). NaH_2_PO_4_•H_2_O (4.1 g) and Na_2_HPO_4_ (1.7 g) were added to the reaction mixture to make up a 0.1 m buffer at pH 7.4. Dithiothreitol (DTT, 2.4 g) was added, and the reaction was stoppered and stirred ON at RT. The resulting crude mixture was dialyzed in 12–14 kDa MWCO RC tubing against 0.1 M NaCl, then water (both corrected to pH 4 using 6 M HCl), before being lyophilized to yield thiolated MC (MC‐SH) as previously described [92], yield = 0.55, and quantified using the Ellman method [90]. Substitution was evaluated at 153 nmol/mg.

#### 5.6.2 XMC Gel Assembly

MC‐SH was reacted with a maleimide (Mal)‐modified binding peptide for the SH3 domain (Mal‐GGGKPPVVKKPHYLS, dissociation constant, K_D_ of 2.7 × 10^−5 ^M) to allow for the affinity‐controlled release of ChASE37 [40, 41, 91]. All materials were sterilized using a 0.22 µm filter. To assemble the hydrogel, unmodified MC, MC‐thiol, and MC‐peptide were combined to achieve a 1:100 molar ratio of protein to binding peptides, and 0.1 µmol thiol per 100 µL of hydrogel in a final concentration of 5% (w/v) MC, within artificial cerebrospinal fluid (aCSF; 149 mm NaCl, 3 mm KCl, 0.8 mm MgCl_2_, 1.4 mm CaCl_2_, 1.5 mm Na_2_HPO_4_, 0.2 mM NaH_2_PO_4_ at pH 7.4). The polymers were dissolved through multiple rounds of speed mixing (35 000 rpm, 2 min) using a SpeedMixer DAC 150 FV2 (FlackTek) and cold centrifugation (13 500 rpm at 4°C, 5 min). Crosslinking was accomplished with 3000 Da PEG‐bismaleimide (Rapp Polymere) at a 0.75:1 maleimide‐to‐thiol molar ratio. Hydrogels were allowed to form ON at 4°C.

For in vivo studies, ChASE37 was added at a concentration of 0.05 U/µL and speed‐mixed into the MC polymer solution. Solutions were kept on ice prior to use.

### 5.7 Hydrogel Characterization

#### 5.7.1 Stability and Degradation

HAO hydrogels (100 µL) were prepared in pre‐weighed Eppendorf tubes and left to gel completely ON at 37°C. Hydrogel mass was recorded before and after swelling with 900 µL of PBS. PBS, hyaluronidase (10 U/mL in PBS, Sigma–Aldrich), or ChASE37 (0.1 U/mL in PBS) were added to the gels. At selected time points, solutions were removed, hydrogel mass was recorded, and fresh PBS or enzyme solutions were added. Stability and degradation ratios were determined based on initial gel mass over a 7 or 28 d period.

XMC hydrogels (100 µL) were prepared in pre‐weighed Eppendorf tubes, speed‐mixed and centrifuged to ensure mixing and then left to settle completely ON at 4°C. Hydrogels were then exposed to 37°C for 1 h to set, prior to the recording of their mass before and after swelling with 900 µL of aCSF. aCSF, cellulase (10 U/mL in aCSF, Sigma–Aldrich), or ChASE37 (0.1 U/mL in aCSF) were added to the gels. At selected time points, solutions were removed, hydrogel mass was recorded, and fresh aCSF or enzyme solutions were added. Stability and degradation ratios were determined based on initial gel mass over a 7 or 28 d period.

#### 5.7.2 Injectability

HOA hydrogels (500 µL) were prepared and promptly loaded into a 1 mL syringe equipped with a 26 G needle tip. The syringe was then affixed to an ISO‐7886‐1 Syringe Compression Fixture, which was mounted on a manual test stand (Model TSB100, Mark‐10). The force required to extrude the gel through the needle tip was recorded over a 2 h period, with 3–5 measurements recorded at each interval.

#### 5.7.3 Mechanical Testing

HAO hydrogels (150 µL) were prepared in 16‐well chamber slides (Nunc Lab‐Tek) and allowed to gel completely ON at 37°C. The following day, the gels were carefully transferred to the platform of a Mach‐1 micromechanical system (Biomomentum), which was controlled by a Universal Motion Controller (Newport). The thickness of the gels was measured first, followed by the determination of their Young's moduli using the stress‐relaxation mode. This involved a ramp amplitude and ramp velocity set to 2% of the initial thickness (with the actual velocity in mm/s calculated individually), and five measurements of 30 s each for relaxation. The Young's modulus was calculated according to Equation (1):

(1)

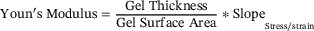




#### 5.7.4 Rheology

Viscoelastic properties were determined using a Discovery HR2 Rheometer (TA Instruments) fitted with a 20 mm steel parallel Peltier plate. Hydrogels (100 µL) were mixed and immediately loaded to the pre‐heated platform. A solvent trap was used to prevent evaporation during the run. Oscillation time sweeps were performed at 37°C, with a 1 Hz frequency, 2% strain, and 30 s sampling intervals for up to 2 h. The rheological data were plotted for shear loss modulus (G″) and shear storage modulus (G′) against time, and the gelation time was determined at the crossover point, where the storage modulus equates to the loss modulus. Measurements were performed in triplicate.

### 5.8 Cell Encapsulation and Imaging

HAO and HAO‐lam hydrogels were synthesized as described above. NPCs were dissociated using Accutase, then centrifuged, reconstituted at a low volume of complete growth media, and counted using Trypan blue exclusion on a CellDrop counter (DeNovix). Media‐containing cells (0.25 × 10^6^) were pipetted into the hydrogel solution, followed by the addition of PEGOA_4_, and immediately dispensed into wells (20 µL, half‐area 96‐well plates, Greiner Bio‐One). Gels were allowed to fully gel at 37°C (1 h), then topped with growth media. Media was replaced twice a week.

Cells were imaged at designated time points using a Spinning Disk Confocal microscope (AxioObserverZ1, Zeiss) across a Z‐axis of 200 µm. Neurite number and length were analyzed on FIJI using the NeuriteJ plugin [93], while clustered cell numbers were quantified using the “analyze particles” method, following binary and watershed processing. At the study endpoint, hydrogels were washed with PBS 3 times, fixed using 4% paraformaldehyde (PFA) for 20 min, and washed again 3x in PBS. ICC was performed as described above. Number of cells and representative images of nuclei distribution in fixed gels were analyzed using Imaris 8 software (Bitplane).

### 5.9 Cell Injection In Vitro

NPCs were dissociated using Accutase, centrifuged, resuspended, and counted. Viability was recorded using Trypan blue exclusion. Approximately 25 000 cells/µL were suspended in an equal volume of either complete growth media or HAO‐lam (final concentration: 0.5% HA). Cell suspensions were loaded into 10 µL Hamilton Gastight syringes equipped with removable 26 G blunt needles. A volume of 2 µL was injected at a controlled rate of 1 µL/min into Eppendorf tubes, and post‐injection viability was immediately assessed via Trypan blue. To evaluate cell survival and differentiation, additional cell suspensions were injected under identical conditions into PLO/laminin‐coated wells. These wells were then topped with differentiation media and cultured for 7 d. Subsequently, the cells were fixed and processed for ICC.

### 5.10 Chondroitinase ABC 37 (ChASE37) Expression and Purification

ChASE37 was expressed and purified as previously described [40], and the construct's complete sequence is available in Hettiaratchi et al. [91]. Briefly, plasmids were transformed into NiCo21(DE3) *E. coli* cells (New England Biolabs) for protein expression. Cells were grown ON at 37°C in LB broth with kanamycin, then transferred to Terrific Broth (TB) with 0.4% glycerol, 50 µg/ml kanamycin, and Antifoam 204 (Sigma–Aldrich). Cultures were aerated in a LEX‐10 bubbler system (Epiphyte3) at 37°C until OD600nm ≥ 0.8, then protein expression was induced with 0.8 mM IPTG (Bioshop) at 22°C ON. Cells were centrifuged at 6000 rpm for 15 min at 4°C (Avanti JXN‐26), and the pellet was either stored at ‐20°C or processed for purification. The pellet was resuspended in 40 mL lysis buffer (50 mm Tris pH 7.5, 500 mm NaCl, 5 mm imidazole, with cOmplete Protease Inhibitor Cocktail (Roche)) and lysed on ice using a 500 W sonicator (QSonica) at 40% amplitude for 5 min at 10 s intervals. The lysate was centrifuged at 12 500 rpm for 30 min at 4°C, and the supernatant was incubated with Ni‐NTA resin (ThermoFisher Scientific) at 4°C for 15 min to encourage binding between the nickel and the His_6_‐tag on the ChASE37 construct. After filtering through a glass column, the resin was washed with 10 × 10 mL wash buffer (50 mm Tris pH 7.5, 500 mm NaCl, 30 mm imidazole). Proteins were eluted with high imidazole concentration (50 mm Tris pH 7.5, 500 mm NaCl, 250 mm imidazole) and concentrated using a Vivaspin 20 concentrator (10 kDa cut‐off, Sartorius). Size exclusion chromatography was performed on a Hi‐load 16/600 Superdex 200 column using a fast protein liquid chromatography (FPLC) instrument (NGC Quest 10 Chromatography System, BioRad Laboratories) in 50 mm sodium acetate, 10 mm phosphate buffer (pH 8.0). All ChASE37‐containing fractions were collected, underwent endotoxin removal with EndoTrap HD 5/1 columns (BioVendor) and sterile filtration (Amicon Ultrafree‐MC 0.22 µm), then concentrated and stored at ‐80°C. Endotoxin levels were measured using the ToxinSensor Chromogenic LAL Endotoxin Assay Kit (GenScript). Protein concentration was determined on an ND‐1000 nano‐drop (Thermo Scientific) by absorbance at 280 nm, using the molecular weight (125 kDa) and extinction coefficient (2.11 × 10^5^ M^−1^ cm^−1^).

### 5.11 Assessment of ChASE37 Activity

The enzymatic activity of ChASE37 was evaluated prior to encapsulation as previously described [40]. Briefly, 10 µL of 0.1 mg/mL ChASE37 was mixed with 90 µL of 10 mg/mL of its substrate, chondroitin sulfate A (CS‐A, Sigma–Aldrich), by measuring the degradation of CS‐A in a UV‐Star microplate (Greiner Bio‐One). Samples were read on a plate reader (Tecan Infinite M200 Pro) at 232 nm for up to 20 min, and the linear slope was used to calculate the kinetic activity of ChASE37 according to the following equation:

(2) $$\mathrm{Activity}\;\;\left(\frac{u}{\mathrm{mg}}\right)=\frac{\mathrm{Slope}\cdot 1U\cdot 10\cdot 60}{\varepsilon \cdot C_{ChASE37}\cdot l}$$

where *U* is enzyme unit $\frac{1\;u\;\cdot min}{\mu mol})$, 10 is the dilution of the enzyme in the assay, 60 is the conversion of seconds to minutes, ε is the extinction coefficient of the soluble product (5100 M^−1^ cm^−1^), *C*_*ChASE*37_ is the concentration of ChASE37, and *l* is the path length (0.32 cm).

### 5.12 Assessment of HAO‐Lam Degradation on Cell Viability

NPCs (0.2 × 10^6^) were seeded on PLO/laminin‐coated wells of a 24‐well plate and allowed to adhere ON. Simultaneously, HAO‐lam gels (50 µL each) were mixed and pipetted into transwells (Greiner Bio‐One ThinCerts, pore size 0.4 µm) and allowed to gel. The following day, baseline viability was recorded using PrestoBlue (Invitrogen), followed by the placement of transwells containing media alone (control), ChASE37 in media (0.1 U/mL), HAO‐lam topped with media, or HAO‐lam + ChASE37 (0.1 U/mL). Viability was measured on days 3 and 6. At the end of the study, HAO‐lam gels remained intact in the transwell, while the disruption of gel integrity was confirmed in the HAO‐lam + ChASE37 group.

### 5.13 Endothelin‐1 (ET‐1) Stroke Model

All experimental procedures were conducted in accordance with the *Guide for the Care and Use of Experimental Animals* and were approved by the Animal Care Committee at the University of Toronto (protocol #20011891). Stroke surgeries were performed following established protocols with minor modifications [40]. Briefly, male Sprague‐Dawley rats (250–300 g, Charles River Laboratories) were anesthetized with 5% isoflurane, and their heads were shaved, cleaned, and secured in a Kopf stereotaxic instrument. Ketoprofen (5 mg/kg) and saline were administered subcutaneously. A rostral‐caudal incision was made along the scalp, and a 2.7 mm burr hole was drilled into the right hemisphere (AP +1.15 mm, ML +3.0 mm relative to bregma) using a trephine drill bit (Fine Science Tools Inc.). To induce stroke injury, 1 µL of 400 pmol/µL ET‐1 (Sigma–Aldrich) was injected at a constant rate of 0.25 µL/min using a 10 µL Hamilton syringe with a 26‐gauge, 45° beveled needle (Hamilton) and an automated microinjector (Harvard Apparatus). ET‐1 was injected at three sites in the motor cortex and striatum: (1) AP 0.0 mm, ML +3.0 mm, DV −2.3 mm; (2) AP +2.3 mm, ML +3.0 mm, DV −2.3 mm; and (3) AP +0.7 mm, ML +3.8 mm, DV −7.0 mm. At each injection site, the needle was allowed to equilibrate for 1 min before starting ET‐1 delivery, and was left in place for 2 min after injection to prevent backflow before being slowly withdrawn. Following injections, a sterile, medical‐grade silicone sheet (Bio‐Plexus, 2.5 mm diameter) was placed over the exposed dura, and the cranial defect was filled with gelfoam (Spongostan Standard) and covered with dental cement (Ortho‐Jet BCA, Lang Dental). The skin was then sutured closed, and the animals were given Ketoprofen and saline before being placed in heated cages to recover. Ketoprofen was given daily for 2 days post‐surgery.

### 5.14 Behavioral Studies

The Grip strength and CatWalk assays were used to assess deficits and functional recovery after stroke. The grip strength assay was used to assess neuromuscular function. During the test, each animal was allowed to grip a T‐shaped bar with its forelimbs, while a handler, who was blinded to the treatment group, gently pulled back on the tail until the rat released its grip. The maximal force exerted by the animal was recorded. Each timepoint was averaged over 4–6 measurements, with 2–3 min of rest between repeats, and presented as a percentage of the individual baseline, normalized to body weight. The CatWalk assay is a gait analysis system used to evaluate motor function in rodents. In this test, each animal traversed a transparent walkway into a dark goal box, while a high‐speed camera captured precise details of each paw's position, pressure, and movement. Animals were trained for 5 d prior to baseline recordings. Each timepoint was the averaged value of 3 successful crossings (speed variation ≤ 60%, run duration ≤ 5 s). Baseline was recorded for all animals 2 d prior to injury.

Animals were randomly assigned into the various treatment groups according to their deficits in the grip strength assay at 4 days post injury (DPI), to ensure similar starting values between groups. Animals lacking a significant deficit from their individual baselines were excluded from the study. Following treatments, animals were assessed on days 10, 14, 21, 28 and 35 post injury.

### 5.15 ChASE37‐AR Delivery and Cell Transplantation

Animals were randomly assigned into one of the five groups (control, vehicle only, NPCs, ChASE37‐AR, or ChASE37‐AR + NPCs, Table 1 and Figure 5A) based on their score in the grip strength assay at 4 DPI.

**TABLE 1:** Treatment groups.

Group	HAO‐lam hydrogel	Encapsulated NPCs	XMC hydrogel	ChASE37‐AR
1. Injury only	—	—	—	—
2. Vehicle only	+	—	+	—
3. NPCs	+	+	+	—
4. ChASE37‐AR	+	—	+	+
5. ChASE37‐AR + NPCs	+	+	+	+

Seven days after injury, rats were anesthetized, dental cement was removed, and tissue was exposed. NPCs (12 500 cells/µL, 50 000 cells/animal) were dispersed in HAO‐lam hydrogel, which was loaded into a 10 µL Hamilton Gastight syringe fitted with a removable 26 G blunt needle. The needle tip was inserted to the same coordinates of the ET‐1 injection (AP 0.0 mm, ML 3.0 mm, DV 2.3 mm) and was allowed to equilibrate for 1 min. HAO‐lam/NPC gels were injected at a rate of 0.5 µL/min (for a total of 4 µL), and the needle was left in place for an additional 2 min before being slowly withdrawn. This procedure was repeated with HAO‐lam gels alone, without NPCs, for the vehicle‐only and ChASE37‐AR‐only groups. XMC hydrogel (6 µL) was loaded with ChASE37 (0.3 U) to form ChASE37‐AR. This was injected epicortically using a 10 µL Hamilton syringe above the lesion site and kept in place and in direct contact with brain tissue using a curved sterile polycarbonate disc, as previously described [40, 49, 94]. ChASE37‐AR was applied in this way for the co‐delivery group with NPCs in HAO‐lam and in the ChASE37‐AR alone group. For the NPCs alone group and the Vehicle group, the process was repeated with XMC hydrogel alone (without ChASE37). For the injury alone control group, an equal volume of saline was injected both into the tissue (instead of HAO‐lam) and in the epicortical space (instead of XMC). Tacrolimus (FK506, Selleckchem) was used as an immunosuppressant and given to all experimental groups. Tacrolimus was dissolved in DMSO, diluted with ethanol and water (final formulation 50:15:35 DMSO:ethanol:water), and given once a day by subcutaneous injections (1 mg/kg body weight) for two days prior to the second surgery. On the day of the second surgery, an osmotic minipump (Alzet, 2ML4) with tacrolimus (1 mg/kg/day) was implanted subcutaneously in all animals. We chose the low dose of 1 mg/kg/day, which was effective and non‐toxic in relevant pre‐clinical studies [95, 96], since high blood levels of tacrolimus can lead to severe side effects, including nephrotoxicity, neurotoxicity, and diabetogenicity. Both incisions were sutured closed, and animals were given ketoprofen (5 mg/kg) and saline before being placed in heated cages to recover. Ketoprofen was given daily for 2 days post‐surgery.

For the preliminary hydrogel administration study, Alexa‐647‐labeled HAO‐lam hydrogel was utilized. The labeling procedure was carried out as previously described [97]. The study was conducted as outlined above. Briefly, 4 µL of the labeled HAO‐lam hydrogel was injected into the lesion site 7 days post‐injury. Animals were sacrificed 2 days later for tissue analysis.

### 5.16 Immunohistochemistry (IHC)

At the study endpoint (35 DPI), animals were sacrificed and transcardially perfused with cold PBS, followed by cold 4% PFA. Brain tissue was subsequently fixed in 4% PFA ON at 4°C, then cryoprotected in a sucrose gradient. The brains were flash‐frozen in cold 2‐methyl‐butane and sectioned coronally at a thickness of 20 µm using a cryostat (Leica CM3050S). Every tenth section was used for staining and quantification.

IHC was performed as previously described [40]. Briefly, slides were allowed to equilibrate to room temperature (RT), then washed 3 times with PBS, followed by permeabilization with 0.3% Triton X‐100 in PBS for 15 min at RT and 3x PBS wash. Slides were then blocked with 10% donkey serum and 0.1% Triton X‐100 in PBS for 1 h, before being incubated ON at 4°C with primary antibodies in the presence of 1% donkey serum and 0.1% Triton X‐100. Details of the antibodies used are provided in Supplemental Table S1. The following day, slides were washed 3x with 0.05% Triton X‐100 in PBS and incubated for 1 h with the appropriate secondary antibodies at RT. Slides were then washed and counterstained with Hoechst 33342 (Cell Signaling) for 10 min at RT, washed 3x in PBS, and mounted using Prolong Gold (Invitrogen). Images were captured using a Zeiss AxioScan.Z1 slide scanner at 20x magnification for quantification and analyzed using FIJI, or a Zeiss LSM 880 confocal microscope at 20x and 63x magnifications for co‐localization analyses.

### 5.17 Quantification of Lesion Volume

Coronal tissue sections spanning the lesion, spaced 400 µm apart, were stained for NeuN and counterstained with Hoechst to quantify lesion volume. The lesion was defined as comprising both the infarct and the cavity. Areas positive for Hoechst staining but devoid of NeuN were classified as the infarct, while regions lacking both Hoechst and NeuN staining were identified as the cavity. Lesion areas were manually traced using Zen software (Zeiss), and the total volume was calculated by averaging the areas of two adjacent sections and multiplying by the distance between them.

### 5.18 Tissue Quantification and Co‐Localization Analysis

To quantify intact and digested CSPGs, microglia, and astrocytes, lesions were identified as regions of interest (ROIs) on Fiji software, based on the absence of NeuN^+^ staining. A pixel threshold was applied to measure areas positive for the appropriate marker, which were then expressed as a fraction of the total lesion area.

To quantify cell fate, ROIs were manually selected based on GFP expression. The co‐localization of GFP with additional markers was analyzed using Zen software, yielding the percentage of co‐localization. The volume of surviving grafts was determined by assessing GFP expression. Similar to the quantification of lesion volume, GFP‐positive areas were measured, and the average area of adjacent sections was multiplied by the distance between them to calculate the graft volume.

### 5.19 Ex Vivo Analysis of FGF‐2 Release Following ChASE37 Treatment

Stroke surgeries were performed as described above. Seven days after stroke induction, animals were euthanized, and brains were rapidly collected and immersed in ice‐cold aCSF. Brains were bisected into ipsilateral and contralateral hemispheres and sectioned into 300 µm‐thick coronal slices in ice‐cold aCSF using a vibratome (LEICA VT 1200S; speed: 0.2 mm/s; amplitude: 1.5 mm). Sections containing the lesion were collected in 24‐well plates, and each section was incubated in 200 µL of either ChASE37 (1 U/mL in aCSF) or aCSF alone (vehicle control) for 1 h at 37°C. Following incubation, supernatants were collected, centrifuged at 3 000 × g for 5 min at 4°C, and stored at −20°C until analyzed. FGF‐2 concentration was quantified by Eve Technologies (Calgary, AB, Canada) using the Human Cytokine/Chemokine Panel A 48‐Plex Discovery Assay on the Luminex 200 platform.

### 5.20 Statistical Analysis

Statistical analyses were determined by PRISM Version 10.4.1 (GraphPad Software Inc.) using one‐way ANOVA with Tukey's multiple comparisons test, a two‐way ANOVA with Šidák's multiple comparisons test, or a t‐test, as appropriate. ^*^ *p* <0.05, ^**^ *p* <0.01, ^***^ *p* <0.001, ^****^ *p* <0.0001. In vivo and tissue analyses data are reported as mean ± standard error of the mean (SEM), whereas in vitro data are reported as mean ± standard deviation (SD) unless otherwise indicated. Illustrations were created using Biorender.com.

## Conflicts of Interest

The authors declare no conflicts of interest and acknowledge a patent for ChASE37 (US Patent Application No. 17/798,415).

## Supporting information




**Supporting File**: advs77213‐sup‐0001‐SuppMat.docx.

## Data Availability

The data that support the findings of this study are available from the corresponding author upon reasonable request.
