# Supplementary material for: Co‐Delivery of Sustained Release Chondroitinase ABC‐37 With Human iPSC‐Derived Neural Progenitors Promotes Transplant Survival and Functional Recovery in a Rodent Model of Stroke
Source: Adv Sci (Weinh). 2026 Sep 9:e22499. Online ahead of print. doi: 10.1002/advs.202522499 (PMC13558902; doi:10.1002/advs.202522499)
Supplement: Supplementary file 1 [file ADVS-9999-e22499-s001.docx]

**Supplementary Information**

**Co-Delivery of Sustained Release Chondroitinase ABC-37 with Human iPSC-Derived Neural Progenitors Promotes Transplant Survival and Functional Recovery in a Rodent Model of Stroke**

Nitzan Letko Khait, David Xinzheyang Li, Keyi Guo, Hong Cui, Quinton Sirianni, Dhana Abdo, Cindi M. Morshead, and Molly S. Shoichet^*^

**Supplemental Figure 1: iPSC differentiation into neural progenitor cells and neurons. (A)** Schematics illustrating the differentiation process. **(B)** qPCR data show the differentiation of iPSCs (day 0) into NPCs (passage 3-4, day 50-60 of neural induction) and neurons (day 28-30 of neuronal differentiation), with the downregulation of the pluripotent marker Oct 3/4, and the upregulation of neural and neuronal markers. Data are reported as mean ± SD, n=3 independent experiments one-way ANOVA with Tukey’s post-hoc, **p<0.01, ***p<0.001, ****p<0.0001. **(C)** Representative images of differentiated NPCs (upper row) and neurons (middle and bottom row), labeled in red/yellow. Scale bar = 100 µm.

**Supplemental Figure 2: NPC encapsulation in HAO hydrogels with increasing laminin concentrations. (A)** Representative images of encapsulated cells in HAO-lam gels with increasing concentrations of laminin. Scale bar = 100 µm. **(B)** The number of cells in the gels is quantified and **(C)** their dispersity (noted as their location on a Z axis) is quantified. Data are reported as mean ± SD, n=4 independent experiments, one-way ANOVA with Tukey’s post-hoc, *p<0.05, ****p<0.0001.

**Supplemental Figure 3: Quantification of NPC number encapsulated in HAO hydrogels with increasing HA concentration.** Data are reported as mean ± SD, n=4 independent experiments, one-way ANOVA with Tukey’s post-hoc, ****p<0.0001.

**Supplemental Figure 4: Rheological characterization of HAO and HAO-lam hydrogels.** As there is no intersection between the storage modulus (G’) and the loss modulus (G”), and G′ > G″ is the accepted rheological definition of a gel, HAO gels can be seen as soft hydrogels immediately upon mixing. Data are reported as mean ± SD, n=3 independent experiments.

**Supplemental Figure 5: HAO-lam administration into the lesion after stroke does not induce additional neuroinflammation.** Expression of Iba1 (microglia) and GFAP (astrocytes) in the infarcted area. Scale bar = 1000 µm.

**Supplemental Figure 6: NPCs maintain high viability and differentiation potential in HAO-lam gels post injection. (A)** Cell viability before and after injection in either media or HAO-lam through a fine needle *in vitro*. Data are reported as mean ± SD, n=3 independent experiments, one-way ANOVA with Tukey’s post-hoc, ****p<0.0001. **(B)** Representative images of injected cells after 7 d of culture. Scale bar = 100 µm.

**Supplemental Figure 7:** Schematic of the reaction between methyl cellulose (MC)-thiol and poly(ethylene glycol) (PEG)-bismaleimide (PEG-MI_2_) to form the crosslinked MC hydrogel (XMC).

**Supplemental Figure 8: Stability and degradation of HAO and XMC hydrogels. (A)** Swelling and enzymatic degradation of HAO in the presence of hyaluronidase (10 U/mL) or ChASE37 (0.1 U/mL) vs phosphate buffered saline (PBS) control. **(B)** Swelling and enzymatic degradation of XMC in the presence of cellulase (10 U/mL) or ChASE37 (0.1 U/mL) vs artificial cerebral spinal fluid, (aCSF) control. **(C)** XMC gel is stable in aCSF for at least 28 d *in vitro.* Data are reported as mean ± SD, n=3 independent experiments.

**Supplemental Figure 9: Animal weight over time. (A+B)** Animals were weighed weekly, and their weights at **(A)** 7 DPI and **(B)** throughout the study are shown compared to their individual pre-injury baseline weights. For **(A)**, data are presented using a min-to-max box plot. Each dot represents one animal, n=8-15 animals per group, one-way ANOVA with Tukey’s post-hoc. ns = not significant, ****p<0.0001. For **(B),** data are reported as mean ± SEM, n=8-15 animals per group, two-way ANOVA with Šidák post hoc, *p<0.05, **p<0.01, ****p<0.0001 (comparison between healthy and all other groups).

**Supplemental Figure 10: ChASE37-AR, NPCs, and their co-delivery, trend towards enhanced recovery in an ET-1 stroke injury model in rats.** **(A+B)** Base of support (BOS) of the **(A)** hind and **(B)** front paws, **(C+D)** stride length of the left **(C)** hind and **(D)** front paws, as measured by the CatWalk assay at 35 DPI. **(E)** Percentage of maximal contact area of the left hind paw, as measured by the CatWalk assay at 14 DPI. n=6-12 animals per group, one-way ANOVA with Tukey’s post-hoc. Data are presented using a min-to-max box plot. Each dot represents one animal. ns = not significant, *p<0.05, **p<0.01.

**Supplemental Figure 11: Transplanted NPCs extend TUBB3^+^ neurites into host tissue.** Representative images of NPC graft, with **(A)** showing overview of graft location, and **(B)** is a zoomed-in image. NPCs extending long neurites into host tissue, co-labeled by GFP and TUBB3, white arrows indicate neurites. Dashed line in (A) indicates graft location. Scale bars = 1000 µm (A) and 20 µm (B).

**Supplemental Figure 12: HAO-lam degrades overtime *in vivo*.** Representative merged image on the left displays hyaluronan, GFP, laminin, and Hoechst, with individual channels on the right. Scale bar = 100 µm.

**Supplemental Figure 13: HAO-lam degradation does not affect cell viability *in vitro*.** Cell viability was measured before and after incubation with transwells containing media alone (control), HAO-lam, ChASE37, or a combination of HAO-lam + ChASE37. Data are reported as mean ± SD, n=3 independent experiments, two-way ANOVA with Tukey’s post-hoc, ns = not significant.

**Supplemental Figure 14: NPCs express GFAP 4 weeks post-transplantation.** Representative merged image (left) display Hoechst, GFP, and GFAP, with individual channels (right) and quantification. Scale bar = 20 µm. n=4 animals, data are presented using a min-to-max box plot. Each dot represents one animal.

**Supplemental Figure 15: Spontaneous differentiation *in vitro*.** A subset of each batch of transplanted cells was plated *in vitro* and cultured for the duration of the study as quality control. **(A)** Cells expressed long extensions and formed connections between clusters. **(B+C)** Cells exhibited spontaneous differentiation, leading to high expression levels of TUBB3 and DCX, while nestin, GFAP, and NeuN expression levels remained low. Quantification of marker co-localization with GFP is shown in **(B)**, while representative images are shown in **(C)**. Data are reported as mean ± SD, n=3-4. Scale bar = 100 µm.

**Supplemental Table 1: IHC and flow antibodies**.

| **Marker** | **Host** | **Dilution** | **Source + catalog #** | **RRID** |
| --- | --- | --- | --- | --- |
| Beta III tubulin (TUBB3) | Mouse | 1:1000 | Abcam ab78078 | AB_2256751 |
| C4S | Mouse | 1:1000 | Millipore MAB2030 | AB_94510 |
| CS56 | Mouse | 1:100 | Sigma Aldrich C8035 | AB_476879 |
| Doublecortin (DCX) | Rabbit | 1:100 | Abcam ab207175 | AB_2894710 |
| GAP43 | Rabbit | 1:500 | Abcam ab16053 | AB_443303 |
| GFAP | Mouse | 1:500 | Cell Signaling 3670 | AB_561049 |
| HuNu | Mouse | 1:500 | Millipore MAB1281 | AB_94090 |
| Iba1 | Rabbit | 1:500 | Wako chemicals 019-19741 | AB_839504 |
| Ki67 | Rabbit | 1:200 | Abcam ab15580 | AB_443209 |
| Laminin | Rabbit | 1:400 | Thermo Fisher PA1-16730 | AB_2133633 |
| Nestin | Rabbit | 1:500 | Abcam ab105389 | AB_10859398 |
| NeuN | Rabbit | 1:500 | Abcam ab177487 | AB_2532109 |
| Oct3/4 | Mouse | 1:200 | BD Biosciences BD611203 | AB_398737 |
| STEM121 | Mouse | 1:500 | Takara Bio Y40410 | AB_2801314 |
| Synapsin-1 | Rabbit | 1:200 | Cell Signaling 5297 | AB_2616578 |
| Synaptophysin | Rabbit | 1:500 | Abcam ab32127 | AB_2286949 |
| TBR1 | Rabbit | 1:500 | Abcam ab31940 | AB_2200219 |
| **Secondary Abs** | **Host** | **Dilution** | **Source + catalog #** |  |
| 488-anti-mouse | Donkey | 1:500 | Invitrogen A21202 | AB_141607 |
| 568-anti-mouse | Donkey | 1:500 | Invitrogen A10037 | AB_11180865 |
| 568-anti-rabbit | Donkey | 1:500 | Invitrogen A10042 | AB_2534017 |
| 647-anti-mouse | Donkey | 1:500 | Invitrogen A31571 | AB_162542 |
| 647-anti-rabbit | Donkey | 1:500 | Invitrogen A31573 | AB_2536183 |
| **Flow Abs** | **Conjugated with** | **Dilution** | **Source + catalog #** |  |
| CD29 (integrin β1) | PE | 1:200 | BioLegend 303003 | AB_314319 |
| CD51 (integrin αv) | PE | 1:200 | BioLegend 327909 | AB_940561 |
| CD61 (integrin β3) | PE | 1:200 | BioLegend 336405 | AB_1227583 |
| CD49e (integrin α5) | PE | 1:200 | BioLegend 328009 | AB_893368 |
| CD49c (integrin α3) | PE | 1:200 | BioLegend 343803 | AB_1731941 |
| CD49f (integrin α6) | PE | 1:200 | BioLegend 313611 | AB_893374 |
| Isotype controls | PE | 1:200 | BioLegend 400507, 400313, 400213, 400111 | AB_326530  AB_2894969  AB_2800438 AB_2847829 |

**Supplemental Table 2: Primers sequences**

| **Primer name** | **Marker for** | **Fw/Rev** | **Sequence (5’→3’)** |
| --- | --- | --- | --- |
| Oct 3/4 | Human pluripotency | Fw | CTGTCTCCGTCACCACTCTG |
|  |  | Rev | TGTGTTCCCAATTCCTTCCTTAG |
| Nestin | Neural progenitor | Fw | AGAAACAGGGCCTACAGAGC |
|  |  | Rev | GAGGGAAGTCTTGGAGCCAC |
| Sox2 | Neural progenitor | Fw | TTGTTCGATCCCAACTTTCC |
|  |  | Rev | ACATGGATTCTCGGCAGACT |
| Pax6 | Cortical/forebrain | Fw | CAATCAAAACGTGTCCAACG |
|  |  | Rev | TGGTATTCTCTCCCCCTCCT |
| Foxg1 | Forebrain | Fw | GCCACAATCTGTCCCTCAACA |
|  |  | Rev | CGGGTCCAGCATCCAGTAG |
| TUBB3 | Neuronal marker | Fw | AACCAGATCGGGGCCAAGTT |
|  |  | Rev | GGCACGTACTTGTGAGAAGAGG |
| Map2 | Neuronal marker | Fw | TCAGAGGCAATGACCTTACC |
|  |  | Rev | GTGGTAGGCTCTTGGTCTTT |
| GFAP | Astrocytic marker | Fw | CAGACCTTCTCCAACCTGCAG |
|  |  | Rev | CTCCTGCTTGGACTCCTTAATG |
| Brn2 | Cortical layer II/III | Fw | CGGCGGATCAAACTGGGATT |
|  |  | Rev | TGCGAGAACACGTTGCCATA |
| Ctip2 | Cortical layer V | Fw | GAGTACTGCGGCAAGGTGTT |
|  |  | Rev | TAGTTGCACAGCTCGCACTT |
| Reelin | Cortical layer I | Fw | ACTGGCATCTTGTCACCGAA |
|  |  | Rev | GTGACAGGCACCCAAAGTTC |
| Tbr1 | Cortical layer VI | Fw | CCGGACAGATGGTGGTTTTA |
|  |  | Rev | GACGGCGATGAACTGAGTCT |
| GAPDH | Housekeeping gene | Fw | AGCAAGAGCACAAGAGGAAGAG |
|  |  | Rev | GAGCACAGGGTACTTTATTGATGG |
